# An NMR-Guided Screening Method for Selective Fragment Docking and Synthesis of a Warhead Inhibitor

**DOI:** 10.3390/molecules21070846

**Published:** 2016-07-16

**Authors:** Ram B. Khattri, Daniel L. Morris, Caroline M. Davis, Stephanie M. Bilinovich, Andrew J. Caras, Matthew J. Panzner, Michael A. Debord, Thomas C. Leeper

**Affiliations:** 1 Department of Chemistry and Biochemistry, The University of Akron, Akron, OH 44325, USA; rbk11@zips.uakron.edu (R.B.K.); dlm93@zips.uakron.edu (D.L.M.); cmd71@zips.uakron.edu (C.M.D.); ajc127@zips.uakron.edu (A.J.C.); mjp9@uakron.edu (M.J.P.); mad151@zips.uakron.edu (M.A.D.); 2 Department of Pathology and Laboratory Medicine, University of North Carolina, Chapel Hill, NC 27599, USA; sbilinovich@gmail.com

**Keywords:** glutaredoxin, FBDD, STD, HSQC, trNOE, warhead, ortholog, docking

## Abstract

Selective hits for the glutaredoxin ortholog of *Brucella melitensis* are determined using STD NMR and verified by trNOE and ^15^N-HSQC titration. The most promising hit, RK207, was docked into the target molecule using a scoring function to compare simulated poses to experimental data. After elucidating possible poses, the hit was further optimized into the lead compound by extension with an electrophilic acrylamide warhead. We believe that focusing on selectivity in this early stage of drug discovery will limit cross-reactivity that might occur with the human ortholog as the lead compound is optimized. Kinetics studies revealed that lead compound **5** modified with an ester group results in higher reactivity than an acrylamide control; however, after modification this compound shows little selectivity for bacterial protein versus the human ortholog. In contrast, hydrolysis of compound **5** to the acid form results in a decrease in the activity of the compound. Together these results suggest that more optimization is warranted for this simple chemical scaffold, and opens the door for discovery of drugs targeted against glutaredoxin proteins—a heretofore untapped reservoir for antibiotic agents.

## 1. Introduction

Fragment-Based Drug Discovery (FBDD) is an emerging method for screening ligands against putative drug target proteins [1]. The goal of the work that follows was applying FBDD techniques to discover and develop a medicinally significant lead molecule with selectivity for a bacterial glutaredoxin while avoiding cross reactivity with the human ortholog. The primary method for discovering prospective leads is a variant of Saturation Transfer Difference (STD) NMR. Positive results were cross-validated via transfer Nuclear Overhauser Effect (trNOE) and Structure Activity Relationship (SAR) by NMR. A library of 463 small fragment compounds was screened for selectivity against two orthologous glutaredoxin proteins, with the rationale that affinity for selective hits may be amplified with chemical synthesis while conserving ortholog specificity. Fragments serve the role of lead molecules to guide development of drug compounds that selectively target the protein of a particular infectious species with reduced likelihood of off-target interactions with host proteins. Although hits obtained from the library are weak (K_D_ > 0.5 mM), they have potential to be chemically linked or elaborated to assemble lead compounds with higher affinity. Such an elaboration has been performed, as demonstrated by synthesis of the chimerical compound RK207ACP.

Acrylamide moieties have been used to functionalize lead compounds by several researchers to develop irreversible inhibitors for some classes of proteins [2,3,4,5]. This acrylamide “warhead” can form a covalent interaction via alkylation with conserved Cys residues in the active sites of these proteins. Although such warheads are often prejudged by medicinal chemists to have poor pharmacokinetics and ADME, recent studies suggest they still have a place in drug development [6]. In particular, linking acrylamide warheads with a specificity-conferring “driving group” can lead to formation of new potent lead compounds [6,7,8]. We suspect that with certain delivery conditions, e.g., nebulization or topical applications, such warhead derivatives may prove quite useful. Cys residues in the conserved active site of target GRXs are expected to react with acrylamide warheads via a Michael addition reaction, in which the warhead vinyl group is the Michael acceptor and the thiolate group of the active site Cys residue is the Michael donor, driving the formation of a covalent adduct [9].

There are several examples of existing drug discovery campaigns focusing on covalent warheads. Using different electrophilic compounds, many research groups are focused on designing irreversible inhibitors for epidermal growth factor receptor (EGFR) [6,10,11,12]. These inhibitors were reported to be effective against secondary mutations associated with cancer cells and thus were promoted to clinical trials [11,13,14]. Cocco and coworkers reported that compounds with an α,β-unsaturated electrophilic warhead showed multi-targeting, anti-pyroptotic activities against the cryopyrin inflammasome regulatory pathway [8]. Similarly, Maresso and coworkers, in search for antibiotic agents, found that compounds containing a β-aminoethyl ketone moiety act as irreversible inhibitors against sortase, a surface-protein-modifying enzyme. Furthermore, aryl forms of these compounds were also reported as irreversible inhibitors for several intercellular kinases. A group at the CHDI foundation reported an acrylamide based irreversible inhibitor that showed high potency and specificity for a transglutaminase enzyme [5]. Similarly, Payne and colleagues developed an acrylamide based compound targeting bacterial enoyl-acyl carrier protein reductase (FabI). This compound displayed an MIC value 500 times lower than commercial antibiotics against the Gram-positive bacterium *S. aureus* [15].

Orthologous glutaredoxin (GRX) proteins from *Brucella melitensis* (BrmGRX) and *Homo sapiens* (hGRX) are the target proteins utilized for screening in this study. The National Institute of Allergy and Infectious Diseases (NIAID) classifies *B. melitensis* as a Category B priority pathogenic organism. *B. melitensis* is known to cause brucellosis and, while in developed countries *Brucella* infection rates are relatively low, it is one of the most significant sources of zoonotic airborne bacterial infections in troubled parts of the world including Syria and to a lesser extent Afghanistan [16,17,18].There are slight differences in amino acid sequences between BrmGRX and hGRX due to evolutionary divergence that result in variations within solvent exposed surface structures of the proteins [19]. This study aims to probe these differences to identify a fragment specific for BrmGRX and to modify that fragment into a lead warhead using an electrophilic group.

Our study is focused on developing an irreversible inhibitor for the GRX ortholog of *B. melitensis*. We chemically synthesized a lead compound containing an acrylamide warhead fused to a fragment hit driving group identified via an NMR-based FBDD assay. The initial fragment hit had selectivity for BrmGRX over hGRX and this subtle selectivity was partially maintained as a warhead functional group was extended into the active site. Selective reaction rates were measured with NMR kinetic assays that determined the modification rates of the GRX orthologs with the control compound (acrylamide) versus ester and acid derivatives of the warhead-fragment chimera. These kinetic data were combined and analyzed to determine the selective reactivity for this new category of irreversible inhibitors.

## 2. Results and Discussion

### 2.1. Library

This study presents the screening of a small library of fragment compounds against two orthologous proteins using STD NMR [20]. Hits were validated with trNOE [21] and ^15^N-HSQC [22]. The library contains 39 scaffolds with different side chains and heteroatoms, the diversity of which results in various binding affinities for different types of proteins. Parameters such as partition coefficients (log *P*), molecular weight, and number of H-bond donors/acceptors in the library were constrained to be adherent to the “rule of three” [4].

### 2.2. Library Screening and Hit Validation

**Figure 1:**
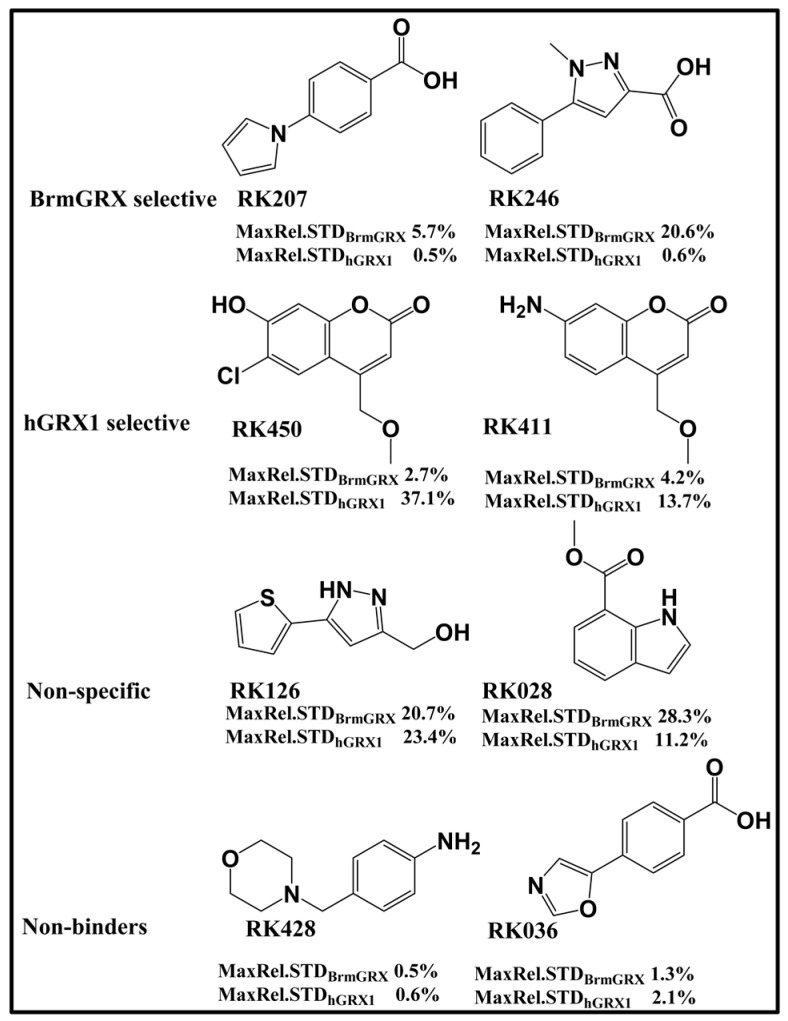
Representative fragments showing different sets of binders.

**Figure 2:**
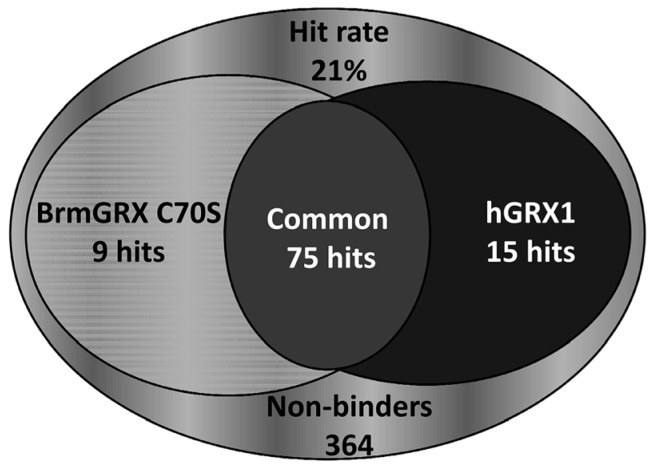
Venn diagram showing STD results. Fragments that interact with one GRX are selective binders. Fragments that bind to both are common binders. Non-binders are fragments that do not interact with either protein.

**Figure 3:**
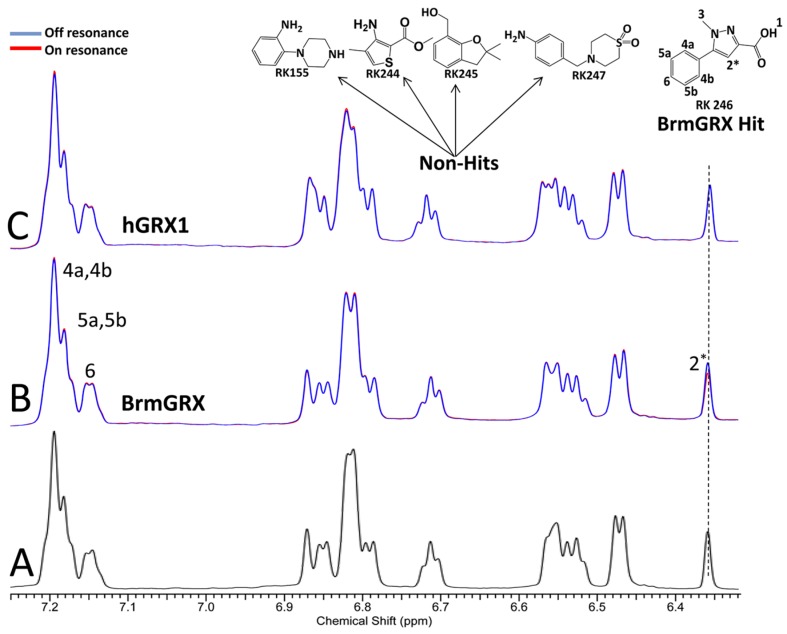
On- and off-resonance STD spectra for a fragment pool containing RK246 against two orthologous proteins. (**A**) The normal ^1^H-NMR spectrum for the fragment pool; (**B**) Shows the STD spectra for the fragment pool with BrmGRX and (**C**) is the STD spectra for the fragment pool with hGRX1. These are collected at 750 MHz with a cold probe at 0.5 mM fragment and 10 μM protein.

STD NMR is a popular method for facile identification of ligand-protein interactions. Its primary advantage over other ligand screening methods is a reduction in the concentration of protein required for analysis with no size limit for the target molecule [20]. Different combinations of selective binders, common binders, and non-binders were obtained from STD screening. Representatives of these sets with relative STD% are shown in Figure 1; a list of fragments is presented in the Supplementary Materials (Figure S1). STD spectra can be collected as separate on and off resonance spectra or as true difference spectra. In this study the former is preferred since they are more convenient for matching peak patterns to reference spectra. The overall STD hit rate was high, with 21% of the fragments interacting with one or both GRXs. The most likely reason for this rate is due to the versatile nature of these small fragments. They have the potential to fit into multiple binding sites because of their size, leading to increased frequency of accommodations [23]. The BrmGRX selective hit rate was only 2%, whereas the hGRX1 selective rate was found to be 3%. Therefore, a more specific subset of compounds is proposed to bind selectively to each GRX. A Venn diagram (Figure 2) compares the set of selective binders, common binders, and non-binders. An example STD spectra of a mixture of fragments measured against both GRXs is shown in Figure 3 (true difference spectra in Figure S2).

RK246 shows a relative STD percentage (rel. STD%) higher than the threshold value for BrmGRX (Figure 1), but not for hGRX1. Therefore, RK246 is considered a BrmGRX-selective hit. The STD spectra for RK207 (the final fragment hit chosen for optimization) against BrmGRX is available in Supplementary Figure S7.

**Figure 4:**
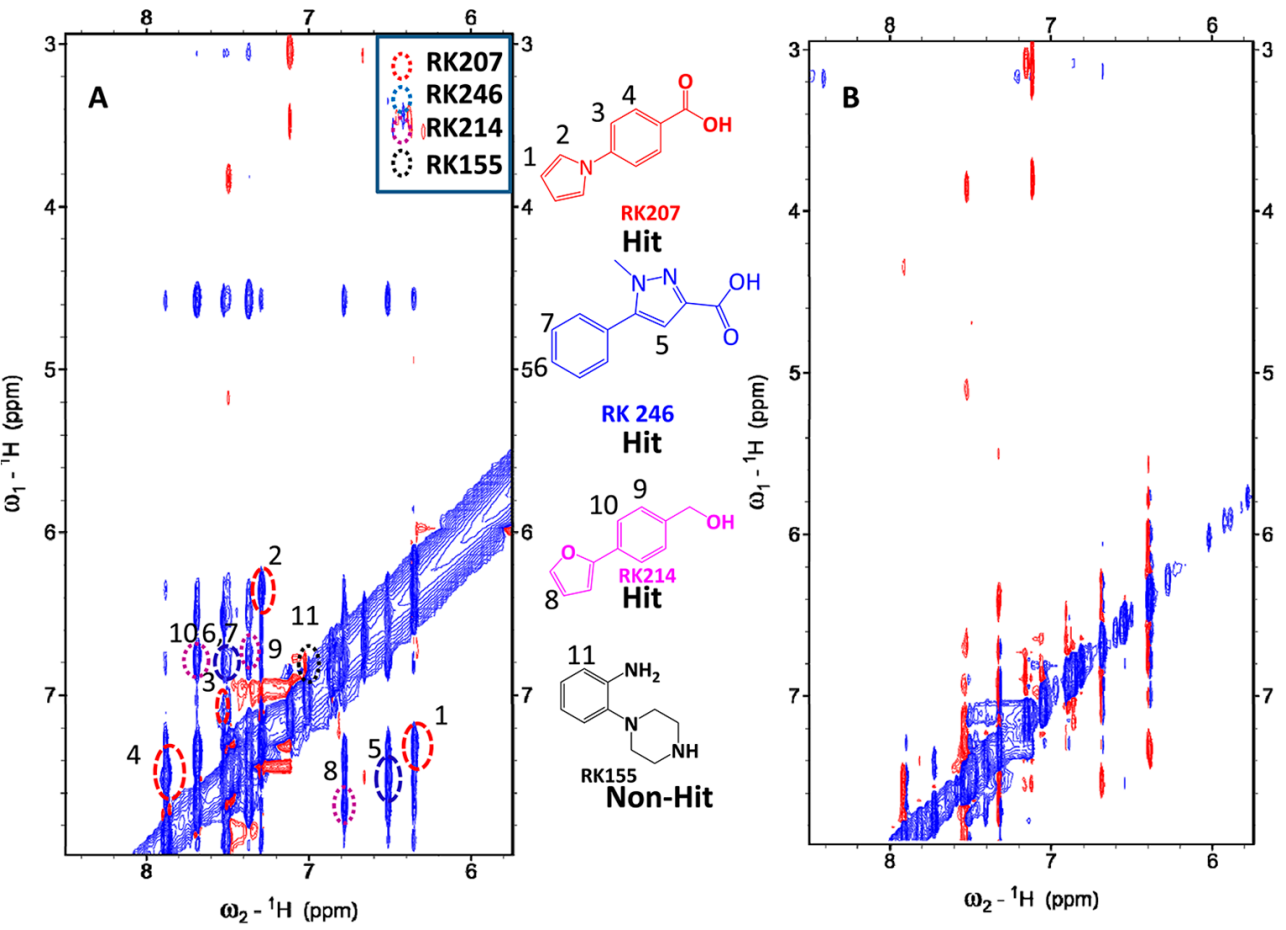
Comparison of the trNOE spectra between the mixture of 0.5mM each of RK207, RK246, RK214, and RK155 with: (**A**) 10 µM of BrmGRX; (**B**) 10 µM of hGRX1. The sign of cross-peaks from bound fragments are the same sign to that of the protein (the sign of the diagonal), thus confirming binding. Peaks corresponding to respective fragment protons are assigned with the same numbers. iLOEs present between fragments as a possible indication of synergistic binding that could be used in future fragment linking studies. Unassigned protons were not picked due to significant signal overlap or failing to appear in the spectrum. Red, blue and purple dotted oval circles on the left spectrum represent peaks for RK207, RK246, and RK214, respectively. As above these spectra are collected at 750 MHz with cold probe.

Selective hits obtained from primary STD screening were further validated beginning with transfer Nuclear Overhauser Effect (trNOE) spectroscopy. The principle of trNOE is that transfer of cross-relaxation between protons can be measured between bound and unbound fragments; this is influenced by the large correlation time of the ligand-target complex via chemical exchange [21]. Upon ligand binding to protein, a large negative NOE in trNOE spectrum is observed [20]. 6% of STD-selective hits for both GRX orthologs were found to show negative NOE peaks in the trNOE spectra, thus doubly confirming some of the STD selective hits to be unique binders with their respective GRX ortholog. Mixtures of hit fragments were used in order to reduce the number of spectra collected (both to conserve NMR time and d_8_ glycerol) and to allow for possible observation of interligand NOEs (iLOEs) useful for the process of linking and merging fagments. These were not considered in the present system but could be used in future fragment growing/linking campaigns. About 55% of BrmGRX selective hits determined using STD were confirmed using trNOE. Comparison of trNOE spectra for fragment mixtures against both GRXs are shown in Figure 4, where fragments RK207, RK214 and RK246 were found to be selective for BrmGRX. RK155 did not bind to either GRX ortholog and was thus considered a “non-hit.” Overlays of the ^15^N-HSQC spectra for RK155, RK214, RK207 and RK246 against BrmGRX are shown in Supplementary Figure S3.

**Figure 5:**
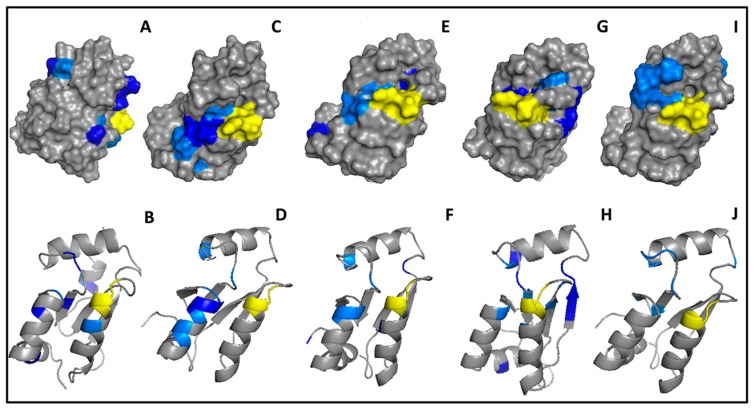
Comparison of binding pockets defined by compounds based on CSPs for BrmGRX and hGRX1. Surface and secondary structures for: (**A**,**B**) RK207 vs. hGRX1, defining multiple possible binding sites; (**C**,**D**) RK207 vs. BrmGRX, having binding pockets near to the conserved CPYS active site; (**E**,**F**) RK207ACPacid [6] vs BrmGRX; (**G**,**H**) RK207ACPester [5] vs. BrmGRX; (**I**,**J**) Acrylamide vs. BrmGRX, respectively (Patches: yellow-CPYC active site; light blue—residues with CSPs exceeding the standard deviation; dark blue—residues exceeding 2× the standard deviation).

Similarly, the entire set of positive hits, including *common* hits, from STD and trNOE primary screening were further validated with ^15^N-HSQC [24]. Among the 99 hits, only 27% induced chemical shift perturbation (CSP) of protein peaks when used in excess concentration (5- to 20-fold). Mapping shifted residues to structure surfaces revealed most hits to bind in the shallow groove present near the active site of GRXs identified previously in a comparative structural analysis [19]. Figure 5 displays the binding pockets occupied by RK207 defined by CSPs upon binding with BrmGRX and hGRX1. RK207 binds in the previously described pocket adjacent to the conserved CPYC active site of BrmGRX when observed at or below a stoichiometric concentration of ligand to protein. However, when an excess of RK207 was used the specificity was lost and the compound further perturbed additional residues in non-specific interactions. Evidence for such interactions is displayed in Supplementary Figure S4. These results clearly suggest that 1D NMR, especially STD NMR, is more sensitive than 2D NMR in the detection of weak fragment interactions with target proteins. In addition, selectivity of some compounds identified during the STD screen were masked at the higher concentrations used for HSQC. Dissociation of the fragment during the mixing time in trNOE may lead to signal loss, making this technique less sensitive than STD NMR [25]. Similarly, lack of strong ring current effects from some fragments may cause low sensitivity observed in ^15^N-HSQC experiments [24,26]. For these reasons, we strongly favor STD as the primary screening method for identifying fragment ligands.

### 2.3. Selection of Hit for Optimization

**Figure 6:**
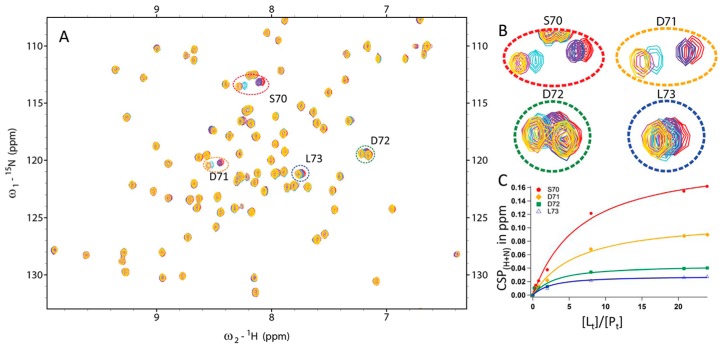
Determination of dissociation constant for ligand-protein complex using 2D NMR. As above these spectra are collected at 750 MHz with a cold probe (**A**) ^15^N-HSQC titration of fragment RK207 against BrmGRX. The red contour is apo, blue is 0.2 mM fragment, purple is 0.5 mM, cyan is 2 mM, magenta is 5.2 mM, and yellow is 6 mM As above these spectra are collected at 750 MHz with cold probe; (**B**) An expanded view of perturbed residues that were used to calculate K_d_; (**C**) Best fit dissociation constant curves obtained from CSP analysis. Curves for residues 70S, 71D, 72D and 73L are shown.

Based on NMR screening results, RK207 was chosen for further study. In spite of its small relSTD%, which is just barely above the threshold for a positive hit, the fragment’s dissociation constant (K_d_) for BrmGRX is 5 to 10-fold better than all other selective hits found. K_d_ values for the best fragments, including RK207 and RK246, were determined using ^15^N-HSQC (Figure 6).

**Figure 7:**
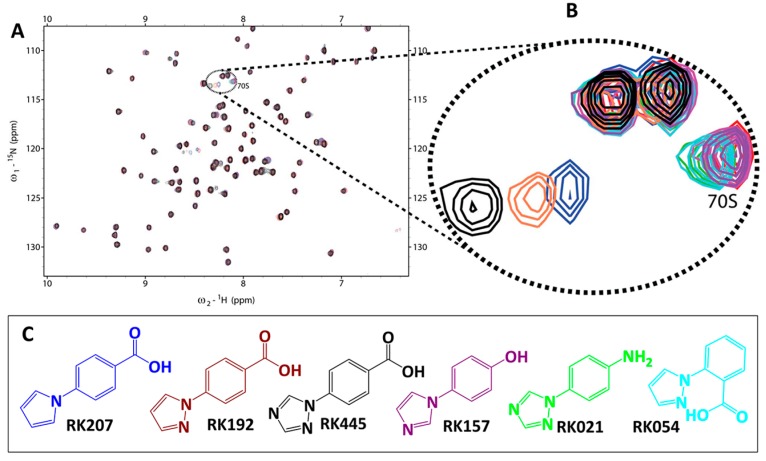
(**A**) ^15^N-HSQC comparison of binding modes among different functional groups as well as their orientation. 2 mM of RK207 and each of its analog fragments are observed against 0.25 mM ^15^N BrmGRX; (**B**) Enlarged S70 peaks; and (**C**) RK207 and its’ analogs. (Red: apo, blue: RK207, brown RK192, black: RK445, magenta: RK157, green: RK021, cyan: RK054).

RK207 was found to be ~2.5-fold more reactive towards BrmGRX than for hGRX1, with K_d_ values of 0.98 ± 0.24 and 2.45 ± 0.52 mM respectively. However, no significant selectivity was observed in RK246 for the BrmGRX ortholog on the basis of HSQC-monitored K_d_ (10.63 ± 5.18 mM for BrmGRX and 5.22 ± 1.16 mM for hGRX1) in spite of its larger apparent STD effects. Several binding experiments were performed using ^15^N-HSQC-NMR for RK207 analogs against BrmGRX. The goal of the post-hoc analysis of the scaffold class was to determine if or how the carboxylic acid moiety of RK207 contributed to these interactions. As shown in Figure 7, RK021, which contains an amide group, shows weaker CSPs than the same fragment with a hydroxyl group (RK157). Large CSPs were observed for fragments containing a carboxyl group, indicating its importance in binding. It was also observed that the orientation of functional groups plays an important role in binding. The significance of carboxyl groups in binding affinity within different classes of proteins has been previously elucidated [27,28].

### 2.4. Fragment Docking for Lead Development

**Figure 8:**
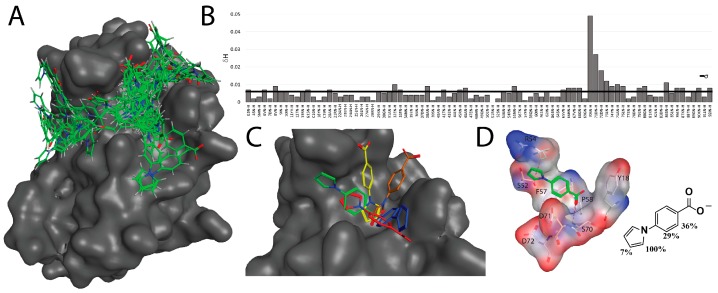
NMR guided docking of RK207. (**A**) Initial docking of 49 possible non-duplicate poses. The BrmGRX surface is rendered in grey; (**B**) Experimental proton shift perturbations. A standard deviation of 0.006 is observed. Experimental and computational shifts below this threshold were set to zero before scoring; (**C**) Computational CSP filtering resulting in these top 5 poses; (**D**) Group epitope mapping comparison of CSP filtered poses reveals a single pose in agreement with STD data. The protein surface is rendered with atom color.

Using molecular dynamics (MD) simulations, computational CSP calculations, and a final STD filter, RK207’s carboxylic acid moiety was predicted to be oriented towards the active site of BrmGRX. MD simulations were carried out in Molecular Operating Environment (MOE) and a database of 48 possible RK207 docked poses were generated [29]. (Figure 8A). Each pose and the apo structure were then run through SHIFTS 5.0.1 and computational CSPs were calculated for backbone amide protons [30]. Predicted shifts were scored against experimental shifts, where computational shifts of the pose that agree most with experimental data is indicative of the pose being measured experimentally. 

Computational prediction of proton chemical shifts has not been routinely used in the past for FBDD projects. McCoy and Wyss first used this method with SHIFTS in 2000 to accurately predict the orientation of a tryptophan-like inhibitor for calmodulin [31,32]. They would later go on to use the same approach in the development of an inhibitor for a hepatitis C protease [33]. Filtering docked poses for fragments on the bases of comparing experimental backbone proton shifts to computationally generated shifts using a P_score_ was only recently validated for FBDD-type screening projects by Aguirre et al. over several manuscripts [34,35,36]. We employed these methods to determine the most accurate binding pose of RK207.

Initial scoring with the CSP filter and using an arbitrary P_score_ threshold of 0.017 resulted in a cluster of the top 5 RK207 poses bound near the α_3_ helix of BrmGRX that lies adjacent to conserved CPYC active site. (Figure 8C) Rel STD% from RK207 STD experiments were used to generate group epitope mapping of the ligand, which agreed with only one pose in this cluster (Figure 8D) .The resulting final pose is primarily stabilized with the double bonded oxygen of the carboxylic acid group in a polar bond with the backbone amide of S70 and the pyrrole ring in a CH–π interaction with an Hγ of R54. Other residues with minor contributions to binding that may also be conferring species selectivity include the sidechains of Y18, S52, F57, P58, G69 and the backbone amide of D71. After examining these results confirming the carboxylic acid moiety oriented toward the active site, chemical elaboration was considered for growing the compound in that direction. Two synthetic routes were considered for optimization. The first route comprised linking this fragment with a ruthenium piano-stool compound and the second route consisted of coupling with an acrylamide moiety.

### 2.5. Initial Warhead-Fragment Strategy: Ruthenium Arene Derivatives

**Figure 9:**
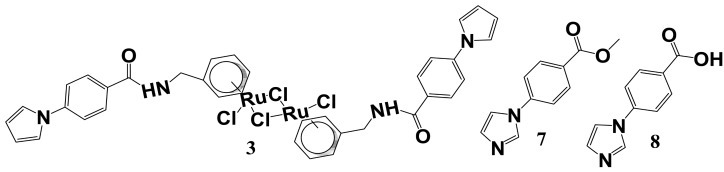
Structures for some synthesized compounds.

The first attempt to couple a warhead to RK207 by adding a ruthenium piano-stool moiety was performed simultaneous to the experiments used for the post-hoc analysis described above. Initial, and somewhat preliminary, docking studies suggested that the carboxylate group of RK207 might be pointing toward the active site. This carboxylate seemed like a convenient coupling group and in the sequence of experiments collected, the post-hoc analysis had not yet determined that it was required for binding. The initial strategy was to couple a ruthenium piano-stool compound derived from benzylamine to RK207, generating an organometallic-based lead compound. Ru-containing compounds have been popular in anti-cancer and antibiotic therapeutic design, as reviewed recently [37,38]. These compounds also tend to interact with soft nucleophilic centers in proteins, such as the thiolates present in GRX active sites [39]. Ru-containing complexes were therefore investigated in their ability to bind to GRX. Early attempts included a complex containing both Ru(III) and RK207. Compound **3** (Figure 9) is an example of such a complex; however, the carboxyl group is involved in an amide bond, rather than being a free moiety. The synthesis scheme for compound **3** is shown in Supplementary Scheme S1. Interactions of the complex with BrmGRX were observed using ^15^N-HSQC, where it was discovered that the RK207-Ru dimer complex did not bind to BrmGRX under ambient temperatures. Lack of binding was likely due to loss of a salt bridge formed between the carboxylate moiety of RK207 and one of the basic residues in BrmGRX, probably R54.

Similarly compound **4**, was also found to be ineffective against BrmGRX when monitored via ^15^N-HSQC because of the unavailability of the carboxyl group to participate at zero NMR time. Compound **7**, which has an ester group, also did not bind with BrmGRX; however, compound **8**, the hydrolyzed acid analog of compound **7** was found to bind to BrmGRX at the zero NMR time. Structure for these compounds is shown in Figure 9 and the synthesis schemes for compound **7** and **8** are shown in schemes 2 and 3 of the synthesis portion of materials and methods part, respectively

Upon further elucidation of the role of the carboxyl moiety in binding to GRX, it was discovered that a negatively charged carboxyl group is required. When binding is observed at a lower pH of 4.5 (just below the pKa value of carboxylic acid), fragments such as RK207, RK445, and RK192 do not bind with BrmGRX. This suggests that at lower pH values, the carboxyl moiety in these fragments becomes protonated and loses its binding capacity, suggesting that modifications at the carboxyl moiety may be deleterious to affinity.

### 2.6. Subsequent Warhead-Fragment Strategy: Acrylamide Derivatives of a Modified Fragment

**Figure 10:**
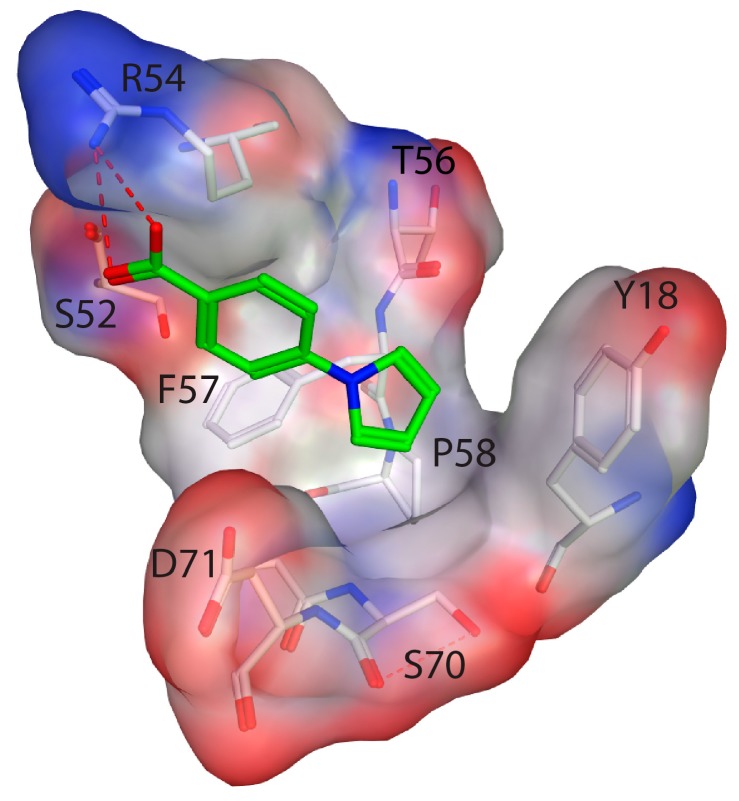
Second ranking pose after CSP and STD filter. The protein surface defining the binding site is rendered with atom color.

Once the importance of the carboxyl group for binding was established, RK207 was modified another way to produce a lead molecule with a preserved carboxyl group and a cysteine-alkylating acrylamide warhead [10]. Another pose of RK207 not in the top-5 cluster, but ranked 13th of the 48 original poses post CSP filtering is oriented in the opposite direction of the winning pose in Figure 8D and is the next highest scoring pose that agrees most with group epitope mapping data (Figure 10).

This pose has RK207’s pyrrole ring oriented towards the active site with the carboxylic acid moiety stabilized by polar interactions with R54’s guanidinium group. Other stabilizing residues include sidechains of Y18, S52, F57, P58 and the backbone amide of D71; all of the same residues stabilizing the best pose save for G69. At this point it was hypothesized that multiple binding modes may be possible and indeed may even be a consequence of species selectivity [40]. The addition of an acrylamide warhead off of the pyrrole ring could still result in a higher affinity binder versus naked acrylamide by increasing residency time in the binding pocket conferred by the RK207 driving group [41]. The nature of warhead covalent inhibitors only requires that they stay in the binding pocket for just enough time for the warhead to react with a target residue before becoming permanently fixed. Despite the sub-optimal pose, an acrylamide warhead off of RK207 pyrrole ring could shift the binding preference of the RK207 driving group to this lower ranked pose.

**Scheme 1:**
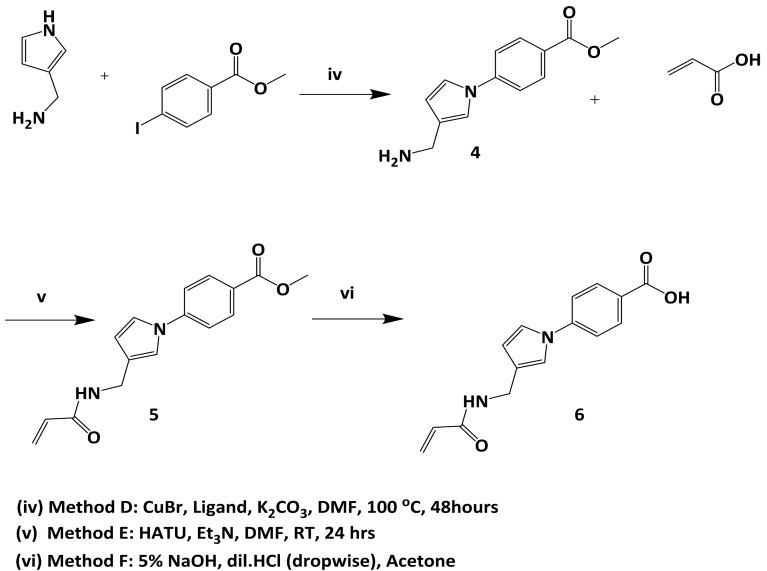
Synthetic scheme for RK207ACP lead compounds.

In the first step, an amine group was introduced to RK207 via copper catalyzed N-arylation of (1*H*-pyrrol-3-yl) methanamine with methyl 4-iodobenzoate in order to facilitate further coupling with acrylic acid (Scheme 1) [42]. This coupling reaction results in the formation of precursor **4** with 20% yield as a white powder. In the second step of the reaction, compound **4** was further coupled with acrylic acid using the standard amide coupling agents HATU and trimethylamine [43]. This reaction resulted in the formation of compound **5** as a yellowish solid with 23% yield. Finally, compound **5** was hydrolyzed with strong base to afford the carboxylic acid compound **6** with 63% yield.

### 2.7. Kinetic Studies and Binding Sites Preferred by Acrylamide Warhead Containing Inhibitor

Kinetic studies were performed with a five-fold excess of compound **5** and compound **6** against reduced ^15^N BrmGRX via ^15^N-HSQC. Rate of inhibited complex formation was inferred by the broadening rate of active site cysteine peaks. Compound **5** showed promising results, taking less than 24 h for the reaction to run to completion (Supplementary Figure S5A). At the zero time-point, there were no CSPs observed for any residues. After an hour of incubation at 25 °C many residues, including the three spectrally visible active site peaks, showed CSPs and/or broadening. Most of these residues lie between the α1 helix and β2 sheet of BrmGRX, near the conserved CPYC active site. These data suggest that the warhead functional group is reacting with the active site, which produces effects on nearby residues. Compound **5** was also found to perturb residues in the α2 and α3 helices (Figure 5). The driving group (the ester form of RK207) accelerates the rate of reaction. Under the same experimental conditions a reaction containing five-fold excess of unlinked acrylamide took more than ten days to reach completion.

A similar study was performed for compound **6**. At the zero time-point, many residues that were associated with the unmodified RK207 binding site showed CSPs, but no changes were observed for the active site residues. Additional residues started to show HSQC chemical shift changes as well as broadening effects after 72 h of incubation at room temperature (Supplementary Figure S5B). From CSPs mapping obtained from ^15^N-HSQC experiment, compound **6** was found to bind in the opposite direction to that of compound **5**, between α1 and α3 helixes near the active site (Figure 5). The reaction runs to completion between 168 to 192 h of incubation at 25 °C. 

Compound **6** was found to be less effective than its ester form. After scrutinizing the CSP map (Figure 5E,F) the most probable reason for this might be formation of an H-bond between the carboxylic acid and S70 or R54, resulting in the compound favoring the primary binding pose oriented in such a way that the warhead is directed away from the active site and remains unavailable for binding with active site cysteine residues. Another probable reason for the lower reactivity of compound **6** as a Michael acceptor is the pH range used in this experiment. Previous studies from several groups showed that at around pH 7, acrylic acids are negatively charged and thus, less reactive than their corresponding esters analogs [44,45].

**Figure 11:**
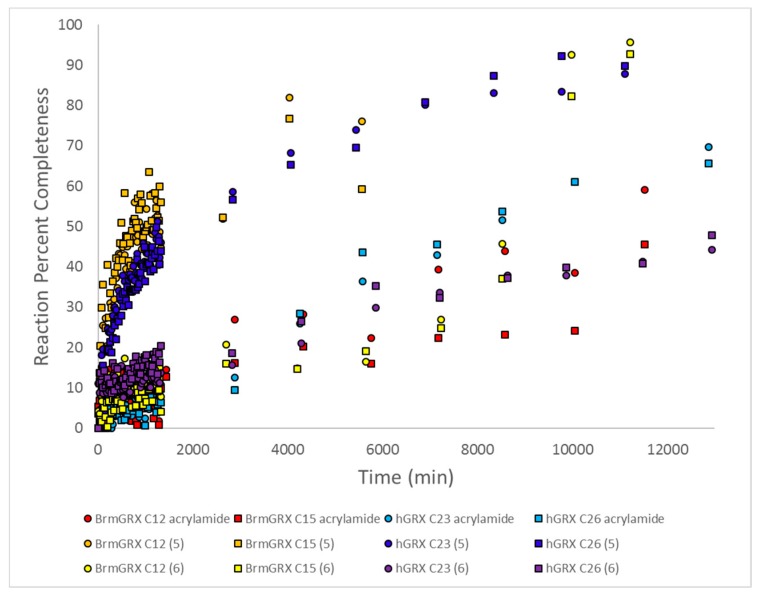
NMR signal-broadening-based kinetic studies for compounds **5** and **6** vs. naked acrylamide. Signal intensity of active site cysteine peaks are normalized to hydrophobic core signals using equation (2) to correct for aggregation and the loss in signal over time is monitored as a quantitative determination of the rate of reaction. The rate was very slow for naked acrylamide and displayed a reaction half life of about 20 h for both proteins. Modifying the ester form of RK207 with an acrylamide warhead (compound **5**) enhances the rate of reaction by about 35 fold for both proteins with a slightly faster rate for BrmGRX. The non-ester form (compound **6**) displays a rate comparable to naked acrylamide.

Quantitative determination of the rate enhancement achieved by linking the driving group with an acrylamide warhead was performed by one to one kinetic studies, monitored via ^15^N-HSQC as before. Kinetics measured under these conditions are more informative because only one lead compound is available per primary binding site, which reduces the likeliness of reaction at multiple binding sites that might occur in the presence of excess compound. Compound **5** was found to be comparatively more reactive than compound **6** for both GRXs. Evaluation of the half-time for the reaction indicated that ester form is 30–35 fold more reactive than its acid analog. The addition of the ester modified RK207 scaffold produces increased residence time of the acrylamide moiety’s vinyl group near the active site of the protein, making it more reactive than plain acrylamide [46,47]. Interestingly, this compound was found to be 1.5–2 fold more selective for BrmGRX (Figure 11).

The probable explanation for this is not only the carboxylic group, but also that the RK207 fragment scaffold plays some role in selectivity and is conserving this selectivity in the lead molecule. Compound **6** was found to have a rate very similar to plain acrylamide. Unfortunately, the inclusion of this acid form driving group makes compound **6** less reactive towards BrmGRX, as suggested by the kinetic plot in Figure 11. The reaction half time of this warhead compound for both BrmGRX and hGRX was similar to plain acrylamide. These data suggest that the inclusion of the acid group in compound **7** does not help compound **8** in increasing reactivity with BrmGRX.

These results were partially corroborated by the bis(2-hydroxyethyl) disulfide (HED) biological assay, where some enhanced inhibition for lead compounds **5** and **6** were observed versus naked acrylamide (Figure S23). The HED assay is a classical assay for GRXs that measures GRX deglutathionylation activity by observing NADPH consumption at 340 nm in a reaction that couples GRX and glutathione reductase. During a preincubation phase GSH and HEDS spontaneously react to form a heterodisulfide. When GRX is introduced it catalyzes deglutathionylation of this disulfide, producing oxidized GSH. As quickly as it is made oxidized GSH is in turn reduced by glutathione reductase which consumes NADPH in a redox reaction [48]. In our hands the HED assay does not give completely reproducible activity with the warhead reaction and we found the concentration of compounds needed to be increased to observe inhibition at a time scale suggested by NMR broadening experiments. This could mean that broadening exhibited in the NMR kinetic experiments is not completely dependent on a covalent reaction and that reduction in active site Cys signal intensity is a result of the compound occupying the active site only. In addition, a two-step serial dilution is required to titrate GRX to HED sample concentrations (10 nM) down from the stock warhead reaction (300 μM). Such a dilution destroys affinity kinetics if the compound has not yet covalently reacted and HED activity likely does not accurately reflect what’s happening in NMR samples. A more systematic approach is warranted and triplicate activity measurements taken over multiple samples will be required to statistically analyze this reaction from a biological assay perspective. Inherently weak binding of these compounds even with addition of the warhead moiety complicates the HED assay and it may prove to be more useful after additional functional group optimization of the leads affords a larger, higher affinity compound to work with.

A mutagenesis approach to the HED assay as well as the NMR experiments is proposed for future work. The active site architecture of BrmGRX matches with the proposed GRX consensus active site structure which suggests the *N*-terminal Cys has the characteristic low pKa associated with GRXs at that residue and should be deprotonated, marking it as the likely active site Cys with warhead vinyl groups [49,50]. Since both active site Cys residues are broadened away in NMR experiments, it is impossible to know which residue is serving as the Michael donor in the warhead reaction. It is possible that both can interact at different population ratios, however covalent linking at either Cys would inhibit activity since both are required to form the disulfide bond necessary for GRX’s reaction mechanism. Nevertheless, the warhead compound may have a higher propensity to react with either Cys and therefore identification of this residue could be important for future compound affinity optimization attempts.

Electrophilic warheads have recently regained researchers’ attention in the drug discovery field [3]. Previous studies have showed that in addition to their use as antineoplastics, warheads can be utilized for developing antibiotics [3,10,13]. While some risks are associated with covalent inhibitors, careful, appropriate compound selection and synthesis may result in potent antibiotic drug candidates [51]. In the search for a lead molecule that is selective towards a bacterial protein, the addition of RK207 as a moderately selective driving group for an acrylamide warhead allowed compound **5** to enhance the rate of reaction by 30–35 fold over naked acrylamide. On the other hand, compound **6** did not contribute greatly in enhancing the rate of reaction. Different functional groups present in these two compounds may orient these molecules differently while binding with BrmGRX, resulting in varying reactivity. Compound **5** seems to have a long residence time in the conserved active site of BrmGRX, leading to early completion of the reaction. Compound **6**, on the other hand, orients itself in such a manner that the warhead is far away from the active site.

Though these results are subtle, carefully chosen linkers with suitable synthesis schemes and several rounds of optimization may lead to a compound that is completely selective towards a bacterial protein. After scrutinizing the kinetic results, it seems that there is a need for more advanced and high throughput technology that provides more precise information about the binding mode and orientation of RK207, so that appropriate linkers can be selected and modified with it to make more effective and bacterial selective leads. In particular, NMR-guided docking will play a significant role in obtaining these poses in a high throughput manner [35]. We believe that covalent protein inhibitors have both balanced benefits and hazards. Appropriately designed experimental protocols will aid in the ultimate success of covalently bound inhibitors in drug discovery. While the selectivity for the bacterial ortholog observed in this study is rather minimal, we believe that these data indicate areas for productive future strategies.

## 3. Materials and Methods

### 3.1. General Information and Library Construction

All bioanalytical grade chemicals utilized were purchased from either Thermo Fisher Scientific (Waltham, MA, USA) or Sigma Aldrich (St. Louis, MO, USA), except when otherwise noted. Warhead lead compounds were prepared in house. The 463-member fragment library was obtained from Maybridge (Cornwall, England) and is a subset of the Ro3 library, which has a Tanimoto similarity index between compounds of less than 0.68 [52,53]. Purity of each fragment was analyzed via 1D ^1^H-NMR. Stock solutions of 100 mM or 200 mM in DMSO-*d*_6_, depending on solubility, were made for long term storage of each fragment. All 1D ^1^H-NMR, STD-NMR, trNOE and ^15^N-HSQC experiments were recorded on an Agilent DD2 750 MHz spectrometer (Agilent Technologies, Santa Clara, CA, USA) equipped with a cryoprobe, in suitable deuterated solvents and at ambient temperature. Electrospray ionization (ESI) and high-resolution mass spectra (HRMS) were taken using a Waters SyNAPT HDMS quadrupole/time-of-flight (Q/ToF) mass spectrometer (Waters, Milford, MA, USA). IR for synthesized lead compounds were taken with a Nicolet iS5 FT-IR Spectrometer (Thermo Fisher Scientific). X-ray crystallography data collected using a Bruker Kappa APEX II Duo CCD system (Bruker, Billerica, MA, USA) armed with a MO ImuS source (λ = 0.71073 Å) and a Cu Imus micro-focus source armed with QUAZAR optics (λ = 1.54178 Å) [54,55,56].

### 3.2. Glutaredoxin Orthologs: Expression and Purification 

Using blastp, it was determined that among the five GRX isoforms found in *Homo sapiens*, hGRX1 shows the highest sequence identity with BrmGRX (38% identity) [57]. The hGRX1 isoform also contains the same conserved active (CPYC) site as the *B. melitensis* ortholog. BrmGRX is a 9.6 kDa protein with 88 amino acid residues whereas hGRX1 is an 11.70 kDa protein with 106 amino acid residues. Cys-to-Ser mutants of both GRX orthologs (C70S for BrmGRX and C83S for hGRX1) were prepared to enhance stability and solubility of these proteins for use at NMR concentrations. All chemical binding analysis was performed with these mutants.

The expression vector containing hGRX1 was purchased from DNA2.0 as a codon optimized derivative of pJexpress 411. The expression vector for BrmGRX was provided by the Seattle Structural Genomics Center for Infectious Disease (SSGCID) as a derivative of pET28a [58]. Each vector contained a T7 polymerase inducible promotor controlling GRX expression. BL21 DE3 competent cells (New England Biolabs, Ipswich, MA, USA) were transformed with each vector respectively and cultures were raised in ^15^N enriched M9 minimal media with shaking at 37 °C until an OD_600_ of ~0.500 was attained. Temperature was then dropped to 18 °C and 0.5 mM Isopropyl β-d-1-thiogalactopyranoside (IPTG) was added to induce protein expression. Cultures were allowed to proceed with induced growth overnight before collection via centrifugation. Cell pellets were re-suspended in affinity chromatography loading buffer, lysed using a French press, and purified using immobilized metal affinity chromatography (IMAC) via a 5 mL HisTrap FF column (GE Healthcare Life Sciences, Pittsburgh, PA, USA). The loading buffer contained 20 mM Tris-Cl (pH 8.0), 200 mM NaCl, 20 mM imidazole and 2 mM dithiothreitol (DTT). Gradient buffer used to elute the purified protein contained a 20-fold higher concentration of imidazole (400 mM), but was otherwise identical in composition to loading buffer. Tobacco Etch Virus (TEV) and HRV 3C proteases were employed to cleave the 6-His tag present on the N-termini of hGRX1 and BrmGRX, respectively. Cleaved proteins were separated from free 6-His tags again using a HisTrap FF column. Purified protein was dialyzed against an NMR buffer consisting of 40 mM sodium phosphate (pH 6.5) and 2 mM DTT. Finally, samples were concentrated using an Amicon stirred cell against a 3 kDa MWCO membrane.

### 3.3. NMR Experiments

#### 3.3.1. STD Screening and trNOE NMR Experiments

STD and trNOE samples contained 10 µM protein, 0.5 mM fragment, 40 mM sodium phosphate buffer (pH 6.5), 2 mM DTT, and 10% deuterated glycerol. These experiments were carried out at 6.2 °C. To decrease the number of samples run, each assay contained a pool of five to seven fragments of diverse scaffolds with non-overlapping ^1^H peaks. All selective hits identified using this method were re-analyzed individually to verify binding with their respective GRX ortholog. Control experiments containing fragments only were also conducted for selective hits to verify purity. Experiments were performed using glycerol and at low temperature to counterbalance the fast tumbling rate of GRXs. These additions cause the protein to tumble slowly, resulting in retardation of rotational correlation time and therefore enhanced cross-saturation efficiency.

In the STD screening experiment, a 50 ms Gaussian-shaped pulse was applied to selectively saturate the methyl protons of well-characterized proteins [20]. The following parameters were used: −14.24 ppm off-resonance frequency, −0.74 ppm on-resonance frequency, 0.682 s acquisition time, 2 s saturation/relaxation period, 64 scans, and 16,000 points. The efficiency of the saturation transfer by the on-resonance frequency on both proteins is shown in Supplementary Figure S6, where STD spectra of proteins only (in upfield region) are shown. To track each sample for aggregation, 1D-NMR was taken before performing STD experiments. These spectra were processed and analyzed using ACD/NMR processing software [59].

The chemical composition of the trNOE sample was consistent with STD samples. The following parameters were used for trNOE: number of points (np) was 2048 with 8 scans, relaxation delay (d1) of 2.1 s, number of increments (ni) was 80 with acquisition time of 0.128 s. The mixing time used was 0.750 s. NMRPipe and NMRFAM-SPARKY were employed for processing trNOE spectra [60,61].

Threshold values were set to parameterize STD percentages that define a fragment as a hit. To be dubbed a “hit,” the fragment must have a Rel. STD% value ≥5% in the downfield region and/or ≥10% in the upfield region. Rel. STD% value was calculated as:

(1) $$\text{Rel}.\text{STD\%}=\;\frac{\left(i_{\text{off}}-i_{\text{on}}\right)}{i_{\text{off}}}\times \;100\text{\%}$$

where *i_off_* and *i_on_* are the intensities of off-resonance and on-resonance peaks, respectively [20,21].

#### 3.3.2. CSP Analysis of Fragment Binding

Amide resonance assignments for orthologous GRXs were retrieved from the Biological Magnetic Resonance Data Bank (BMRB) [62]. Structures of target proteins were obtained from the Protein Data Bank (PDB) [63]. The PBD code for BrmGRX is 2KHP and hGRX1 is 1JHB [19,64]. Sample composition for ^15^N-HSQC experiments consists of the following: glutaredoxin (0.25 to 0.5 mM), 40 mM sodium phosphate buffer pH 6.5, 5% D_2_O, 2 mM DTT, and 0–5 mM fragment. All spectra were again processed and analyzed using NMRPipe and NMRFAM-SPARKY.

Kinetic studies were performed by incubating 0.3 mM of reduced protein with 0.3 mM of compound and monitoring broadening of active site cysteine peaks as a function of time as the rate of reaction. Intensity of active site cysteine peaks were normalized against a hydrophobic core glycine to correct for contributions to broadening from aggregation. ^15^N-HSQC spectra were collected in succession at 25 min intervals for the first 1300 min of the reaction. Further time points were taken as instrument time was available until complete broadening away of active site peaks was observed. The percent completion of the reaction as monitored by loss of signal in the active site was normalized for aggregation using Equation (2):

(2) $$\text{\% Completeness}=100\times \left[\frac{\left({\frac{\text{Cys}}{\text{Gly}}}^{\text{max}}-{\frac{\text{Cys}}{\text{Gly}}}^{t_{x}}\right)}{{\frac{\text{Cys}}{\text{Gly}}}^{\text{max}}}\right]$$

where, $\frac{\text{Cys}}{\text{Gly}}=\frac{I_{{\text{Cys}}_{t_{x}}}}{I_{{\text{Gly}}_{t_{x}}}}$. In this equation ${\frac{\text{Cys}}{\text{Gly}}}^{\text{max}}$ is the maximum peak intensity ratio of an individual active site cysteine relative to a hydrophobic core glycine over all time points. ${\frac{\text{Cys}}{\text{Gly}}}^{t_{x}}$ is the same ratio taken at time point *x*. The peak intensity ratio is calculated as $\frac{I_{{\text{Cys}}_{t_{x}}}}{I_{{\text{Gly}}_{t_{x}}}}$ where the numerator and denominator are the intensity of the active site cysteine being measured and the intensity of the hydrophobic core glycine respectively. G63 and G66 were used for BrmGRX and hGRX respectively.

Dissociation constants (K_d_) for selected fragments were determined using titrations with increasing fragment concentrations while maintaining a constant glutaredoxin concentration. A non-linear regression (Equation (3)) was employed to calculate K_d_ [65,66]. Igor Pro 6.36 was used to fit the data [67]. Here, CSP_(H+N)_ is the total Euclidean distance traveled in both hydrogen and nitrogen dimensions and is scaled by Equation (4):

(3) $${\text{CSP}}_{\left(H+N\right)}={\text{CSP}}_{\text{max}}\frac{P_{t}+L_{t}+K_{d}-\sqrt{{\left(P_{t}+L_{t}+K_{d}\right)}^{2}-4L_{t}P_{t}}}{2P_{t}}$$

(4) $${\text{CSP}}_{\left(H+N\right)}=\sqrt{\frac{1}{2}\left[\delta_{H}^{2}+\left(\alpha \times \delta_{N}^{2}\right)\right]}$$

Values of α were 0.2 for glycines and 0.14 for all other residues [68]. CSP_max_ is the maximum CSP that results from saturation. P_t_ and L_t_ represents the overall concentrations of the target protein and hit compound used, respectively. Ligand efficiency (LE) was calculated using Equation (5) [69,70]:

(5) $$L.E.=\frac{\Delta G}{N}=-\mathrm{RT}\;\ln\;K_{d}$$

where, ΔG is Gibbs’ free energy, N represents the number of heavy atoms present in the hit, R is gas constant and equal to 8.314 J·K^−1^·mol^−1^, T is the absolute temperature.

### 3.4. Computational Studies

#### 3.4.1. Ligand Docking 

Prior to docking, hot spots were predicted using the FTMap web server with BrmGRX’s NMR solution structure (PDB: 2KHP) [19]. FTMap takes PDB structures as input and then samples billions of probe positions on translational and rotatable grids using the Fast Fourier Transform method. The 16 default small organic probe molecule were used. Areas on the protein surface where probes minimize and overlap are termed consensus clusters (CC) where the cluster with the largest number of probes in considered to be the primary hot spot. CCs were then extracted and superimposed onto the original apo structure. CCs that were not within reasonable proximity to residues identified by CSP analysis were removed, leaving CCs defining a large binding pocket partially occupied by GSH under physiological conditions [71] (Figure S22). Docking was conducted with the organic solvent molecules of the CCs serving to define the binding pocket, partially filling the role of a crystallographic ligand used in most site-directed docking simulations.

All docking experiments were conducted and visualized within Molecular Operating Environment (MOE). A stochastic conformational library of the conjugate base form of RK207 was generated and used to sample initial binding poses. A flattened structure of RK207 was imported into MOE and partial charges were calculated before minimization in an explicit water droplet using the AMBER12-EHT force field. A conformational search was then carried out on the molecule using MOE’s automatic stochastic search function, resulting in a four conformation library used to sample initial docking poses [72]. The 2KHP structure was prepared using MOE’s automatic structure preparation after using the homology modeling tool to simulate a C70S mutation. Charges on chain termini were corrected and missing hydrogens were added before a final minimization using the same force field as above. 20,000 initial poses were sampled using triangle matcher placement, which is exhaustive for small molecules [73]. Poses were ranked using the London dG scoring function and duplicates were removed [29]. Remaining poses were refined using a rigid receptor protocol and rescored using MOE’s GBVI/WSA dG function before final duplicate removal [73]. A database of 48 final poses bound to the apo structure was generated and each model was exported to an AMBER formatted PDB file.

#### 3.4.2. SHIFTS Simulations for Computational CSPs

Simulated proton chemical shifts were calculated using SHIFTS similarly to the McCoy and Wyss method. Only contributions to theoretical shift from ring current effects of the ligand were considered, where a ring binding at the surface of a protein can induce CSPs in atoms 7–10 Å away [31]. Nitrogen chemical shifts are not calculated due to lack of acceptable models [30,74,75]. SHIFTS calculates ring-current effects on proton shifts based on the Haigh-Maillon semi-classical model [76]. In SHIFTS simulations the benzene and pyrrole ring effects from RK207 are calculated independently of one another. McCoy and Wyss used a tryptophan based inhibitor where they set the rings of their ligand to be parameterized as a tryptophan sidechain in SHIFTS. In our method the benzene ring was set to parameters for a phenylalanine sidechain and the pyrrole ring was set to parameters from one ring in a heme cofactor. The heme pyrrole ring based simulation is acceptable because SHIFTS breaks apart cofactor rings in simulations in the same way the benzene and pyrrole are separated in ours.

To compare simulated pose shifts to experimental data and filter out the dominating poses, the P_score_ function (Equation (6)) proposed by Aguirrie et al. was employeed [34]. This function is a normalized version of the Q_score_ originally used by McCoy and Wyss [31]:

(6) $$P_{\text{score}}=\frac{1}{N}\overset{}{\underset{i}{\sum }}{\left(\frac{{\text{CSP}}_{\text{exp}}\left(i\right)}{{\text{CSP}}_{\text{exp}}^{\text{max}}}-\frac{{\text{CSP}}_{\text{calc}}\left(i\right)}{{\text{CSP}}_{\text{calc}}^{\text{max}}}\right)}^{2}$$

here, a lower P_score_ indicates docked positions that are in good agreement with CSPs observed in experimental data. N is the number of observable non-proline residues, ${\text{CSP}}_{\text{exp}}\left(i\right)$ is the experimental proton CSP observed at residue i, ${\text{CSP}}_{\text{calc}}\left(i\right)$ is the experimental proton CSP observed at residue i, and ${\text{CSP}}_{\text{exp}}^{\text{max}}$ and ${\text{CSP}}_{\text{calc}}^{\text{max}}$ are the largest experimental and simulated CSPs observed over all residues, respectively.

### 3.5. Synthesis

#### 3.5.1. Preparation of Methyl 4-(1*H*-imidazol-1-yl) Benzoate or RK464

**Scheme 2:**
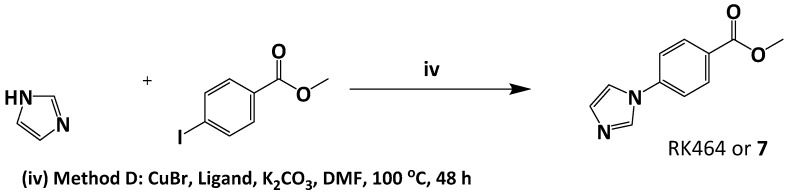
Synthetic scheme for compound **7** or RK464.

For the synthesis of RK464 (Scheme 2), a round bottom flask with a magnetic stir bar was used as a reaction vessel. To this vessel, CuBr (7.2 mg, 0.025 mmol), (1*R*,2*R*)-*N*1,*N*2-dimethyl- cyclohexane-1,2-diamine (16 µL, 0.05 mmol), and imidazole (82 mg, 0.6 mmol), were added with DMF (20 mL) as a solvent. The vessel was sealed with a septum and nitrogen gas was passed through it.

After reacting the initial mixture of compounds, methyl 4-iodobenzoate (262 mg, 0.5 mmol) and K_2_CO_3_ (357 mg, 2.6 mmol) were added. Again, nitrogen gas was back-filled and the reaction mixture was heated in a preheated oil bath for 48 h at constant temperature of 100 °C. Reaction progress was regularly monitored using TLC. As the reaction progressed, the color of the mixture changed from white to yellow. The product was extracted using ethyl acetate and purified using flash chromatography. A pure yellow viscous product (about 40 mg) was obtained and dried under vacuum. Yield: 19%. ^1^H-NMR (750 MHz, CD_3_OD) δ: 6.75 (s, 1H, Ar-H), 6.63–6.64 (d, 2H, Ar-H), 6.19-6.20 (d, 2H, Ar-H), 6.16 (s, 1H, Ar-H), 2.40 (s, 3H, CH_3_) (Supplementary Figure S9); HRMS (ESI) calcd C_11_H_10_N_2_O_2_ for 203.0821, found 203.0808 [M+H]^+^, (Supplementary Figure S14). FT IR ν_max_ cm^−1^: 1710.61 (carboxyl, C=O), (Supplementary Figure S20). X-ray crystallography: X-ray crystallography structure for this compound is shown in Supplementary Figure S19 with R = 0.2. 

#### 3.5.2. Preparation of 4-(1*H*-*imidazol*-1-yl)benzoic acid or RK465 

**Scheme 3:**
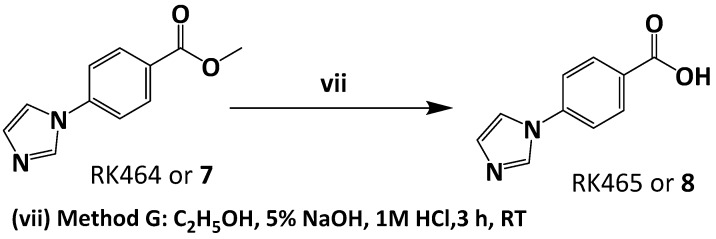
Synthetic scheme for compound **8** or RK465.

In a round-bottom flask, 20 mg of compound **7** was dissolved in 4 mL of ethanol and stirred with 2 mL of 5% NaOH solution for 2 h (Scheme 3). Formation of the hydrolyzed product was regularly monitored using TLC.

After 2 h, the reaction appeared close to completion as suggested by TLC. At this point, 1 M HCl was added dropwise until the mixture turned acidic (monitored with litmus paper). A white product precipitated after roto-vaporization. The product was soluble in methanol, leaving behind NaCl crystals. The product was dried under vacuum, resulting in a white powder. Yield: 90%. ^1^H-NMR (750 MHz, CD_3_OD) δ 9.61 (s, 1H, Ar-H), 8.25–8.26 (d, 2H, Ar-H), 8.18 (s, 1H, Ar-H), 7.88-7.90 (d, 2H, Ar-H), 7.82 (s, 1H, Ar-H), (Supplementary Figure S10). FT IR ν_max_ cm^−1^: 1715.44 (carboxyl, C=O), (Supplementary Figure S21). HRMS: calculated for C_10_H_8_N_2_O_2_ 189.0664, found 189.0672 [M + H]^+^, (Supplementary Figure S15).

#### 3.5.3. Preparation of 4-(3-(Acrylamidomethyl)-1*H*-pyrrol-1-yl)benzoate (**4**)

To a round bottom flask was added CuBr (7.2 mg), (1*R*,2*R*)-*N*1,*N*2-dimethyl- cyclohexane-1,2-diamine (16 µL), and (1*H*-pyrrol-3-yl) methanamine (58 mg). The mixture was sealed with septum, evacuated, and back-filled with nitrogen gas. Methyl 4-iodobenzoate (262 mg) and K_2_CO_3_ (357 mg) were then added. DMF (20 mL) was used as a solvent. Sodium carbonate was preferred because using potassium hydroxide (a hard base) resulted in hydrolysis of the ester moiety of the product. The ligand to copper ratio was 2:1. The mixture was heated for 2 days at 100 °C. Reaction progress was monitored via TLC. Ethyl acetate was used to extract the organic phase from the aqueous layer. Flash chromatography was used to purify the product. The white-colored product was eluted with a mixture of ethyl acetate to hexane with a yield of 20%. ^1^H-NMR (750 MHz, CDCl_3_) δ 8.37 (s, 1H, NH), 7.85–7.86 (d, 2H, Ar-H), 7.11 (s, 1H, Ar-H), 6.76 (s, 1H, Ar-H), 6.58–6.59 (d, 2H, Ar-H), 6.22 (s, 1H, Ar-H), 4.23 (s, 2H, CH_2_, 3.84 (s, 3H, CH_3_), (Supplementary Figure S11). HRMS: calculated for C_12_H_12_N_2_O_2_ 253.0953, found 253.0936 [M + Na]^+^, (Supplementary Figure S16).

#### 3.5.4. Preparation of Methyl 4-(3-(Acrylamidomethyl)-1*H*-pyrrol-1-yl)benzoate (**5**)

In a round bottom flask, 1-[bis(dimethylamino)methylene]-1*H*-1,2,3-triazolo[4,5-b] pyridinium 3-oxid hexafluorophosphate (HATU, 102 mg), triethylamine (Et_3_N, 40 µL), and acrylic acid (12 µL) were taken with DMF (5 mL). The mixture was sealed with septum, evacuated, and back-filled with nitrogen gas. After 15 min, 30 mg of compound **4** was added to the mixture. Again, the flask was back-filled with nitrogen gas for 1 h and stirred for 48 h at room temperature. A yellowish solid compound was formed with a yield of 23%. ^1^H-NMR (750 MHz, CDCl_3_) δ: 8.63 (s, 1H, NH), 8.08–8.09 (d, 2H, Ar-H), 7.87 (s, 1H, Ar-H), 7.40–7.41 (d, 2H, Ar-H), 7.12 (s, 1H, Ar-H), 6.78–6.79 (d, 1H, -C=CH-), 6.59–6.63 (m, 2H, Ar-H & H_2_C=C-), 6.36 (s, 1H, H_2_C=C-), 4.28 (s, 2H, CH_2_), 3.92 (s, 3H, CH_3_) (Supplementary Figure S12). HRMS: calculated for C_16_H_16_N_2_O_3_ 307.1059, found 307.1058 [M + Na]^+^ (Supplementary Figure S17A,B). 

#### 3.5.5. Preparation of 4-(3-(Acrylamidomethyl)-1*H*-pyrrol-1-yl)benzoic acid (**6**)

Compound **5** (10 mg) was hydrolyzed to form the acid derivative **6**. The mixture was dissolved in 3 mL of acetone and stirred with 2 mL of 5% NaOH for 3 h. Formation of product was monitored with TLC. HCl (1 M) was added dropwise until an acidic pH was reached. A white precipitate was formed and purified via filtration. ^1^H-NMR (750 MHz, CDCl_3_) δ: 8.02 (s, 1H, NH), 7.85–7.90 (d, 2H, Ar-H), 7.39 (s, 1H, Ar-H), 7.22–7.28 (d, 2H, Ar-H), 7.11 (s, 1H, Ar-H), 6.80–6.81 (d, 1H, -C=CH-), 6.61–6.65 (m, 2H, Ar-H & H_2_C=C- ), 6.50 (s, 1H, H_2_C=C-), 4.31 (s, 2H, CH_2_) (Supplementary Figure S13). HRMS: calculated for C_15_H_14_N_2_O_3_ 269.0926, found 269.0911 [M − H]^−^ (Supplementary Figure S18).

### 3.6. HED Assay

Warhead mediated inhibition of GRX activity was monitored using the HED assay, originally proposed by Nagai and Black [77]. The protocol here was adapted from Zaffagnini et al. with the only modification of doubling the concentration of glutathione reductase (GR) [78]. This was done because we experienced GR being a rate limiting reagent when attempting to run trials with GRX concentrations above 10 nM. Briefly, a reaction mixture containing 0.7 mM HEDS, 0.1 mg/mL BSA, 1 mM reduced GSH, 12 µg/mL glutathione reductase, and 0.2 mM NADPH was assembled in 100 mM Tris-Cl, pH 7.9. The reaction mixture is separated equally into a sample and reference cuvette and after a 3-minute incubation to facilitate the spontaneous formation of the GSH-HED mixed dosulfide, 10 nM GRX is added to the sample cuvette alongside an equal amount of buffer to the sample cuvette. Activity was monitored by the GR catalyzed oxidation of NADPH at 340 nm as it reduces the oxidized glutathione produced by GRX using a Shimadzu UV-1800 spectrophotometer. GRX activity is isolated by observing the change in absorbance at 340 nm in the sample cuvette versus the reference cuvette as a subtraction from the baseline NADPH oxidation rate. GRX activity was calculated from the first 60 s of each reaction and activity was expressed at mM of NADPH oxidized/min.

Sample preparation for the HED assays was slightly different compared with set-up for the NMR experiments. Initial attempts at a 1:1 stoichiometric ratio of compound to protein produced little reduction in activity in early time points. In an effort to speed up the reaction and see inhibition at the beginning of the reaction a ratio of 4:1 compound to protein was selected moving forward. This requires a compound concentration of 1.2 mM which unfortunately was not soluble under 100% aqueous conditions. Therefore, stock reactions for the HEDS assay were set up with 12% DMSO to facilitate solubility of the compounds at 1.2 mM. Otherwise the reactions were set up the same as NME kinetic studies with 40 mM sodium-phosphate buffer at pH 6.5, 20 mM NaCl, 0.3 mM GRX, and 1.2 mM compound. For Figure S20, four reactions were started at the same time with compound **5**, compound **6**, acrylamide, and an apo reaction to which only DMSO was added to 12%. Activity was taken twice a day for 4 days.

## 4. Conclusions

This study presents the design, synthesis and development of a covalent inhibitor for a bacterial protein using the principles of FBDD. A small library was screened and validated with NMR methods for two GRX orthologs. Most of the hits obtained were found to show low millimolar selectivity for the bacterial GRX. A majority of the hits were versatile in nature, binding with both GRXs. The best hit (RK207), was submitted to an NMR guided docking routine to elucidate fragment binding poses. An ester modification of this RK207 driving group coupled with an acrylamide warhead enhanced the rate of reaction by 30 to 35-fold with small selectivity for the bacterial protein, while the acid form of this compound does not enhance reactivity. These results may inspire future projects to develop more selective covalent inhibitors for bacterial GRXs by optimizing the driving group with an appropriate linker.

## Supplementary Materials

Supplementary materials can be accessed at: http://www.mdpi.com/1420-3049/21/7/846/s1.

## Abbreviations

The following abbreviations are used in this manuscript:

FBDD: fragment-based drug discovery
STD: saturation transfer difference
Relative STD%: relative saturation transfer difference in percentage
NMR: nuclear magnetic resonance
HSQC: heteronuclear single quantum correlation
SAR: structure activity relationship
BrmGRX: *Brucella melitensis* glutaredoxin
hGRX1: human glutaredoxin 1
*E. coli*: *Escherichia coli*
HTS: high throughput screening
log P: log of partition-coefficient
K_d_: dissociation constant
L.E.: ligand efficiency
CSPs: chemical shift perturbations
trNOE: transferred nuclear Overhauser effect
DMSO: dimethyl sulfoxide
D_2_O: deuterium oxide
DTT: dithiothreitol
NaCl: sodium chloride
Et3N: triethyl amine
HATU: 1-[Bis(dimethylamino)methylene]-1*H*-1,2,3-triazolo[4,5-*b*]pyridinium 3-oxid hexafluorophosphate
