# Supplementary material for: An NMR-Guided Screening Method for Selective Fragment Docking and Synthesis of a Warhead Inhibitor
Source: Molecules. 2016 Jul 16;21(7):846. doi: 10.3390/molecules21070846 (PMC6274284; doi:10.3390/molecules21070846)
Supplement: Supplementary file 1 [file molecules-21-00846-s001.pdf]

# Supplementary Materials: An NMR-Guided Screening Method for Selective Fragment Docking and Synthesis of a Warhead Inhibitor

**Ram B. Khattri, Daniel L. Morris, Caroline M. Davis, Stephanie M. Bilinovich, Andrew J. Caras, Matthew J. Panzner, Michael A. Debord and Thomas C. Leeper**

- **Common hits for both proteins:**

  RK088, RK149, RK028, RK391, RK209, RK381, RK266, RK204, RK347, RK188, RK445, RK384, RK174, RK061, RK318, RK397, RK172, RK132, RK276, RK118, RK142, RK224, RK371, RK462, RK220, RK114, RK423, RK063, RK179, RK044, RK334, RK227, RK304, RK451, RK337, RK320, RK098, RK262, RK440, RK439, RK419, RK093, RK310, RK033, RK018, RK201, RK458, RK086, RK147, RK202, RK238, RK306, RK291, RK400, RK405, RK407, RK447, RK409, RK301, RK163, RK463, RK006, RK218, RK242, RK344, RK386, RK066, RK126, RK005, RK054, RK010, RK041, RK399, RK208, RK200

  Total = 75

- **BrmGRX selective:**

  RK197, RK207, RK214, RK196, RK246, RK032, RK144, RK215, RK275

  Total = 09

- **hGRX1 selective:**

  RK104, RK112, RK333, RK374, RK211, RK129, RK156, RK182, RK183, RK236, RK192, RK272, RK411, RK412, RK450

  Total = 15

**Figure S1.** Primary STD screening results showing the sets of common binders and selective binders. Red colored fragments passed additional validation with trNOE.

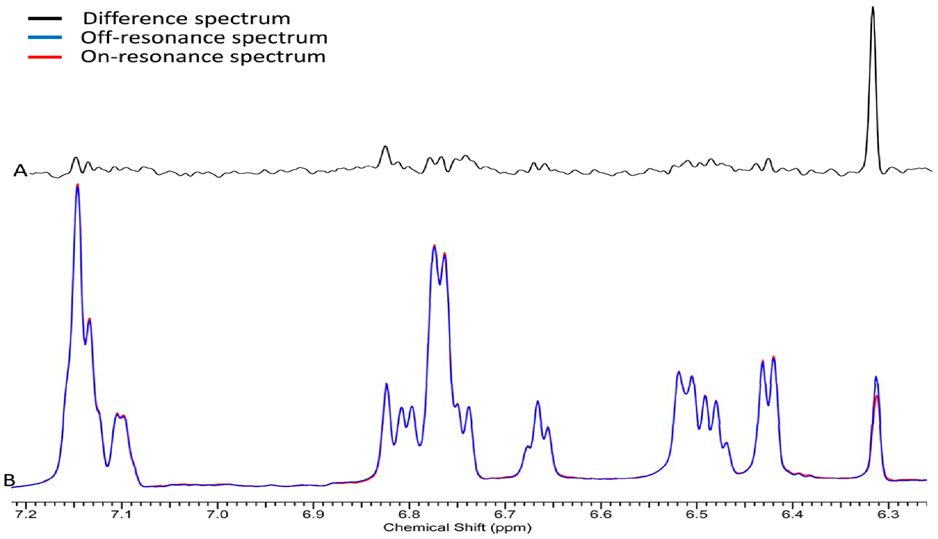


**Figure S2.** Different types of STD spectra. A) Diffference spectrum for a mixture of fragments against BrmGRX, B) Off-resonance spectrum (blue) and on-resonance spectrum (red) for the same set of fragments (as with difference spectrum) against BrmGRX.

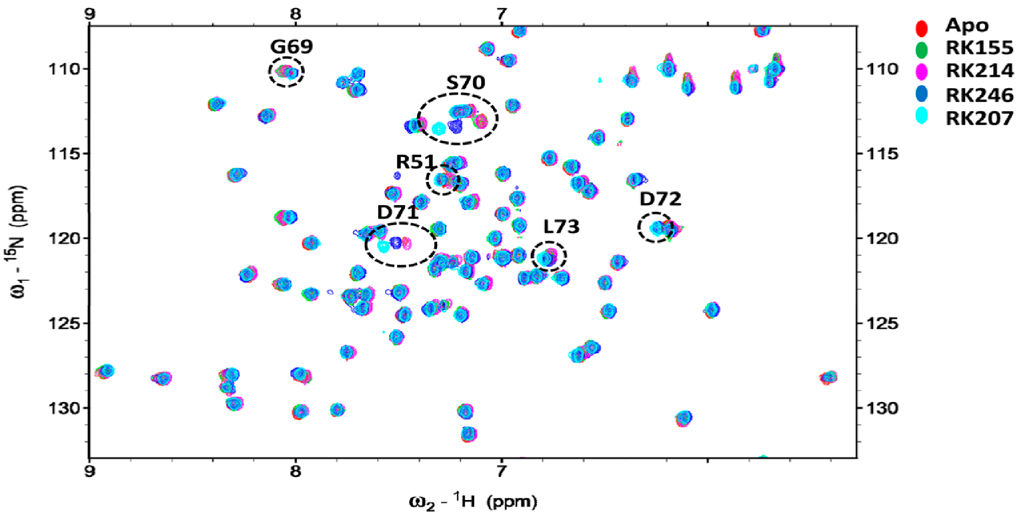


**Figure S3.** Overlays of the $^{15}$N-HSQC spectra for RK155, RK214, RK207, and RK246 against BrmGRX. RK207 showed maximum CSPs.

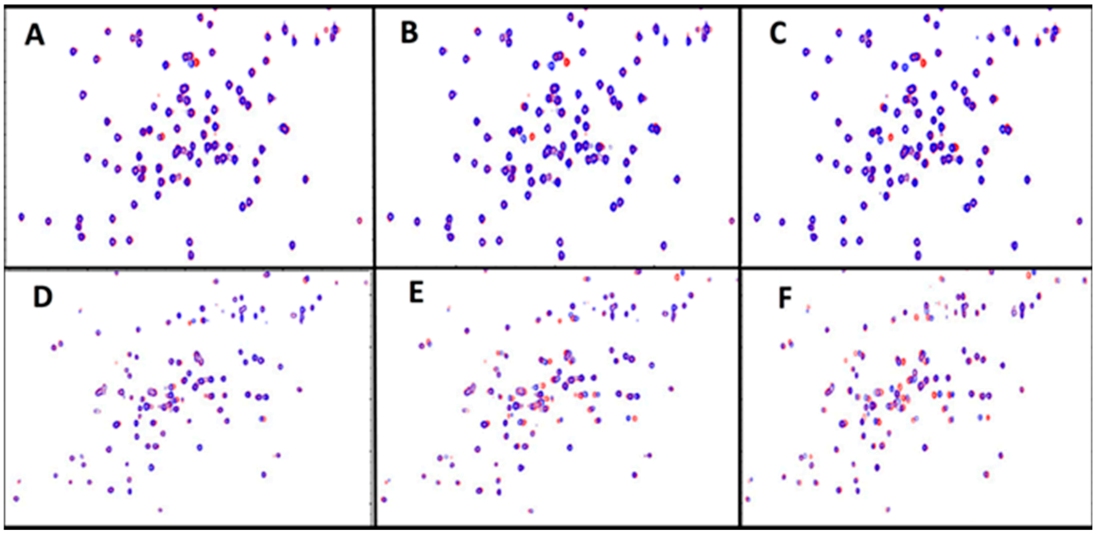


**Figure S4.** $^{15}$N-HSQC experiments for fragment RK207 against orthologous proteins showing concentration dependent specificity. (**A**), (**B**), and (**C**) represents $^{15}$N-HSQC spectra of 0.25 mM BrmGRX with 0.5 mM, 2 mM and 5 mM of RK207, respectively. Similarly, (**D**), (**E**), and (**F**) for 0.25 mM hGRX1 with 0.5 mM, 2 mM and 5 mM of RK207, respectively. (Red- apo, blue- with fragment).

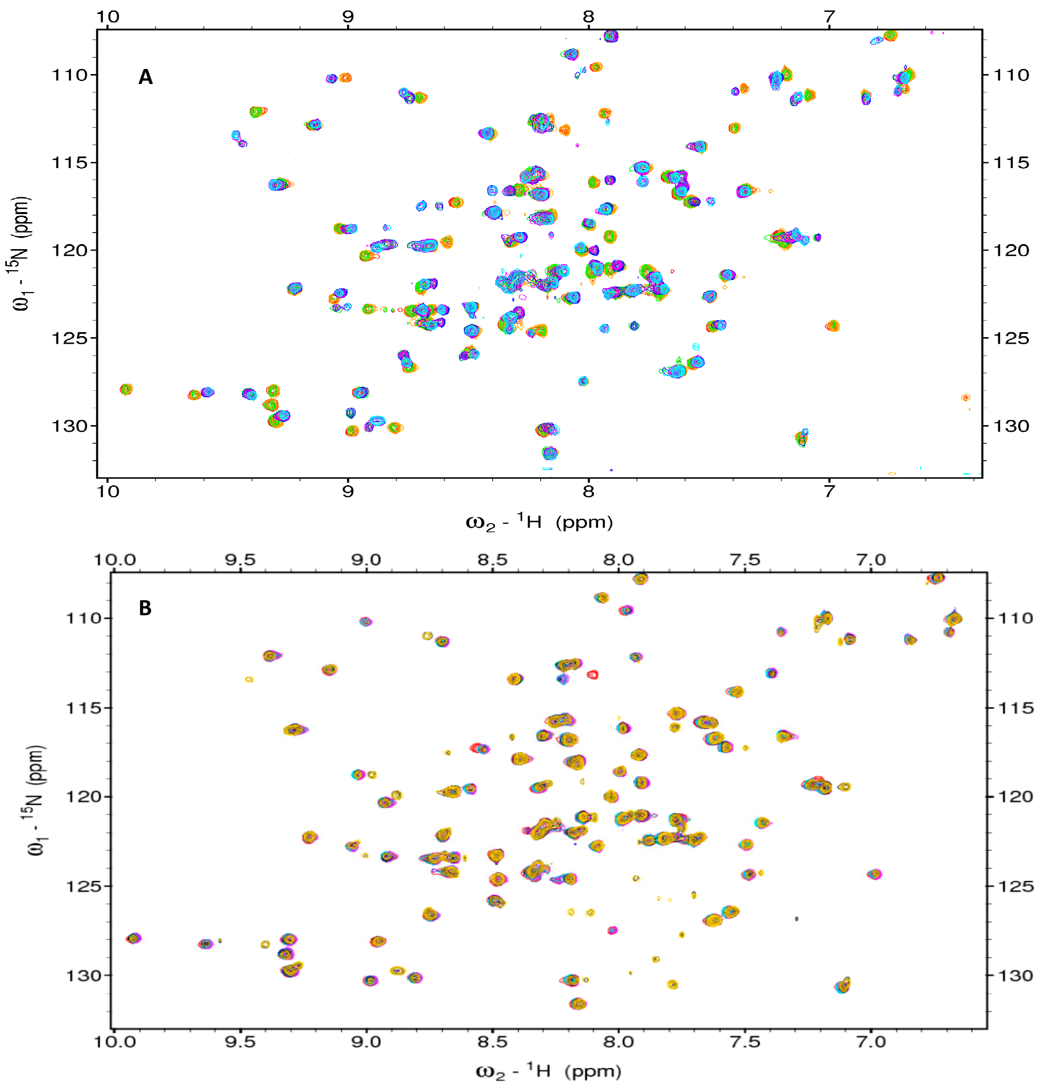


**Figure S5.** Overlays of spectra for kinetic studies of 5 fold excess of compound against BrmGRX performed via $^{15}$N-HSQC at different time intervals. **(A)** Compound **5** vs BrmGRX, Red: Apo; Incubation periods at 25 °C with compound:- orange: zero NMR time, green: 1 h, blue: 2 h, magenta: 6 h, and cyan: 24 h. **(B)** Compound **6** vs BrmGRX, Red: Apo, Incubation periods at 25 °C with compound:- orange: zero NMR time, green: 1 h, blue: 2 h, Tomato red: 6 h, magenta: 24 h, cyan: 48 h, purple: 96 h, Black: 168 h, and Gold: 192 h.

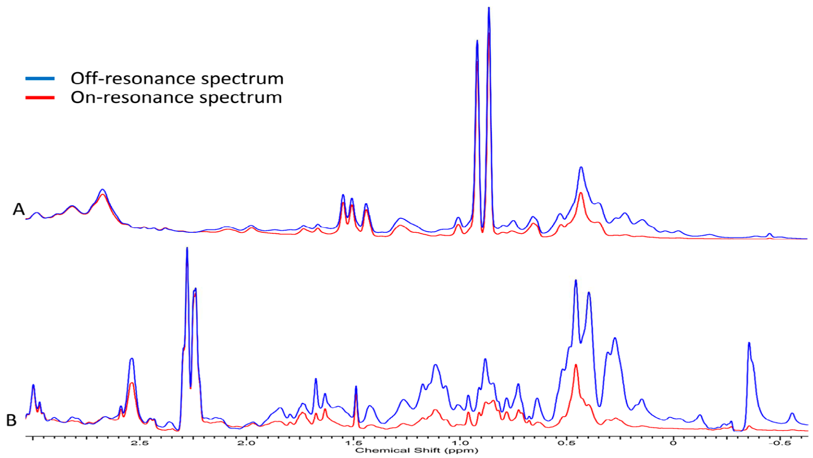


**Figure S6.** STD spectra of proteins without fragments (upfield region). A) STD spectrum for hGRX1 protein, B) STD spectrum for BrmGRX. Off-resonance and on-resonance frequency applied were −14.24 ppm and −0.74 ppm respectively, for both proteins. The protein concentration used was 0.2 mM in deuterated environment.

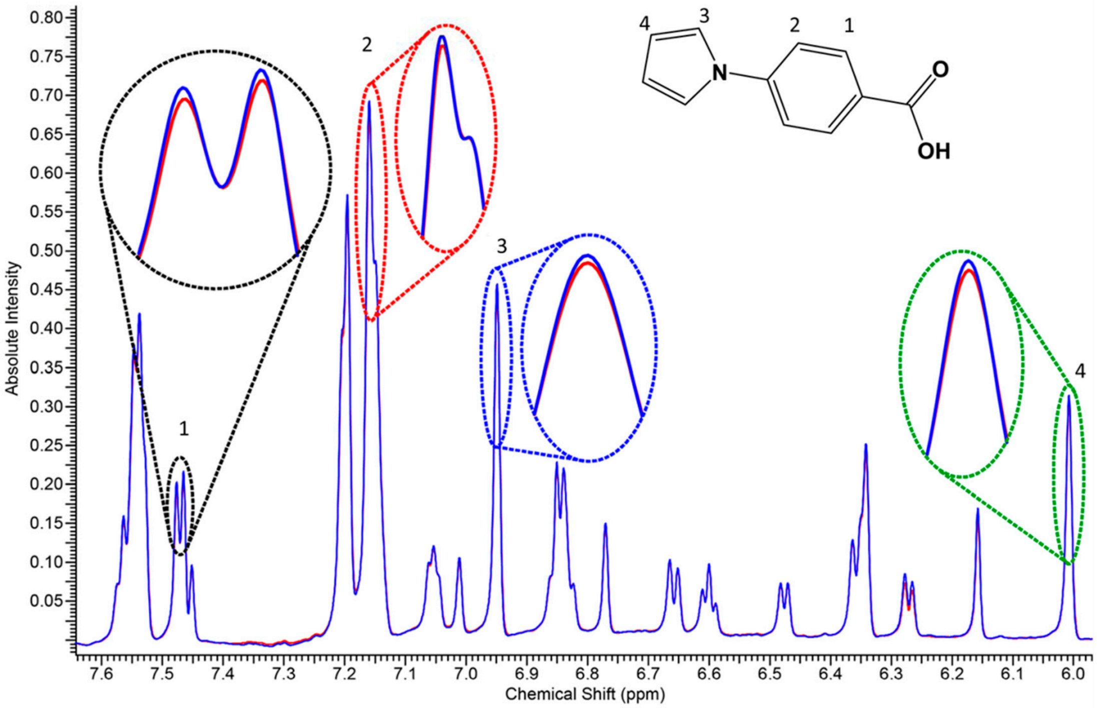


**Figure S7.** STD spectra for RK207 (in mixture) against BrmGRX. The expanded view for each of the aromatic protons of RK207 is also shown.

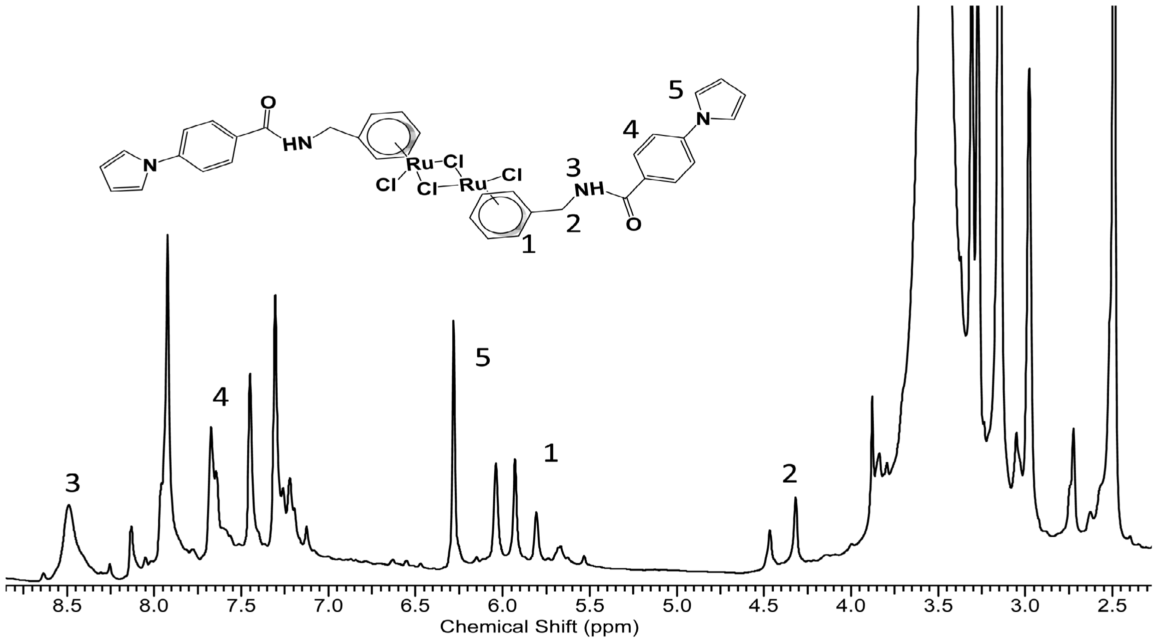


**Figure S8.** $^{1}$H-NMR spectrum for compound **3**. Upfield –shift occurred for aromatic hydrogens because of the interaction with ruthenium. Some impurities signals are present, which are from solvents and starting materials. $^{1}$H-NMR (DMSO) δ: 8.65 (s, 1H; N-H), 7.21–8.02 (multiple peaks; 4H; Ar-H), 6.30 (s, 4H, pyrrol), 5.78–5.86 (multiple peaks; 5H; Ar-H), 5.56 (s, 1H; cyclohexadienyl H), 3.81 (s, 2H; N-CH2), 2.61 (t; 2H;NH-C-CH2-CH-CH-), 1.09 (t, 2H; CH-CH2-CH) ppm.

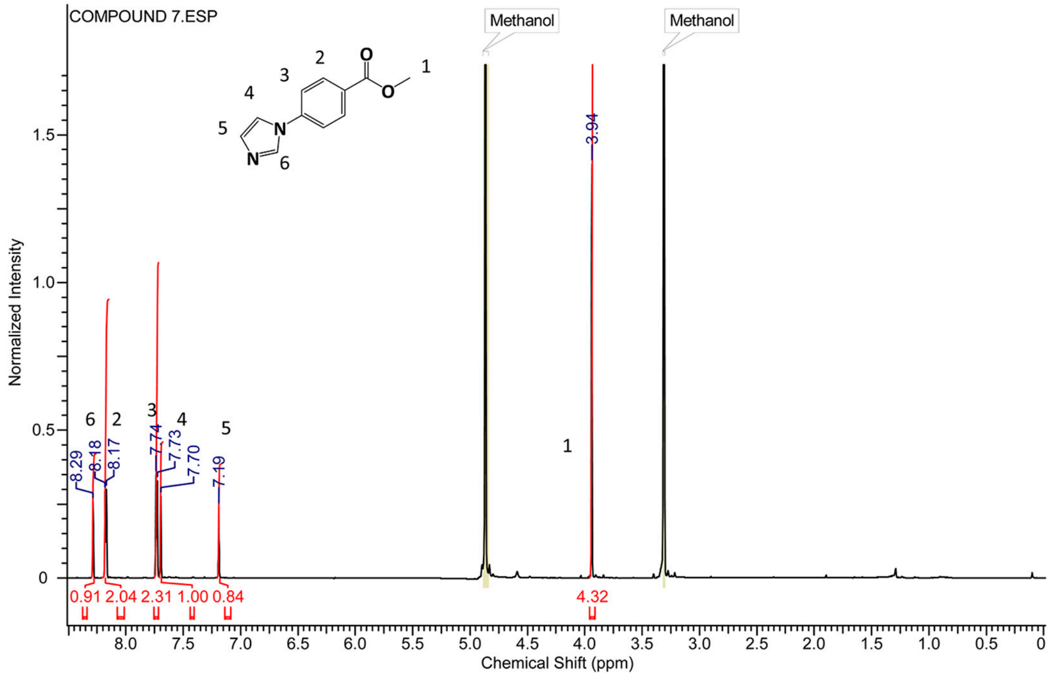


**Figure S9.** $^{1}$H-NMR spectrum for compound **7** or RK464 in deuterated methanol. This compound was further verified with HRMS, IR and X-ray crystallography. The thermal ellipsoid structure of this compound is shown in Figure S19.

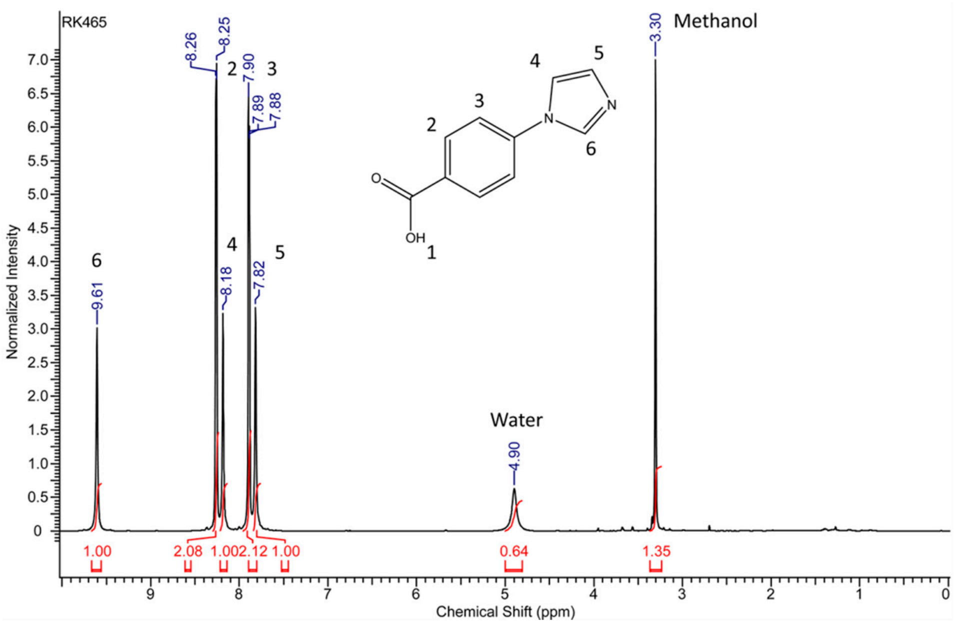


**Figure S10.** $^1$H-NMR for compound **8** or RK465 in deuterated methanol.

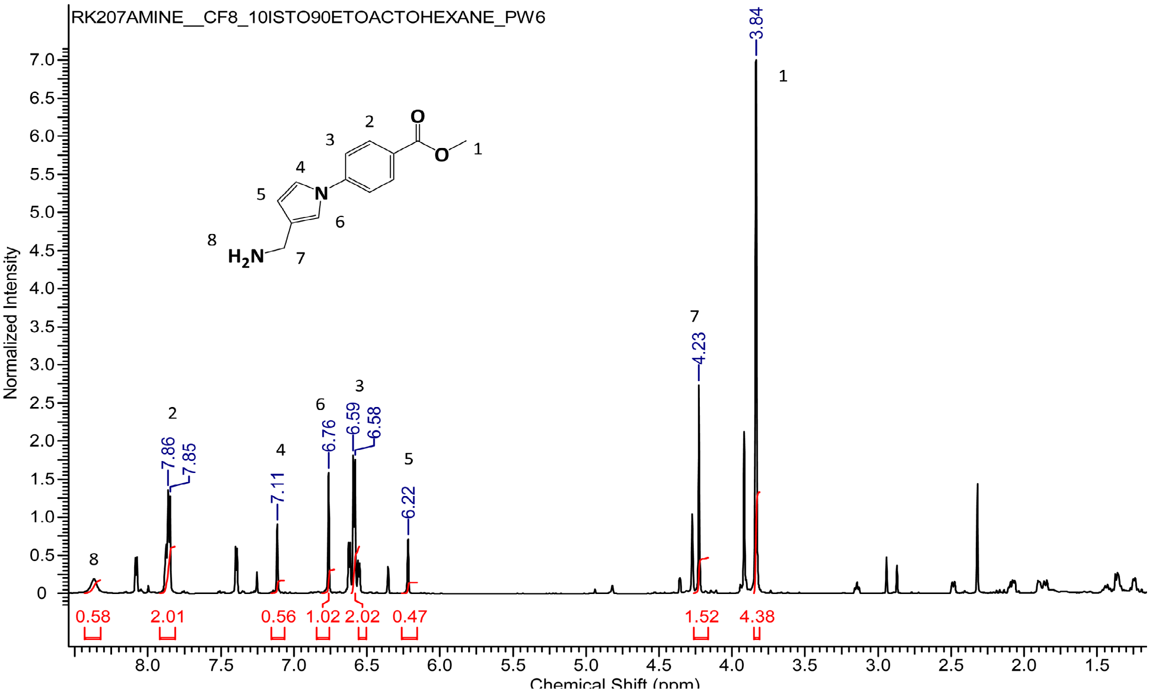


**Figure S11.** $^1$H-NMR spectrum of compound **4.** Some impurities signals from starting material such as methyl 4-iodo benzoate and solvents can be seen. The estimated relative percentage purity of this compound was found to be about 77% by moles.

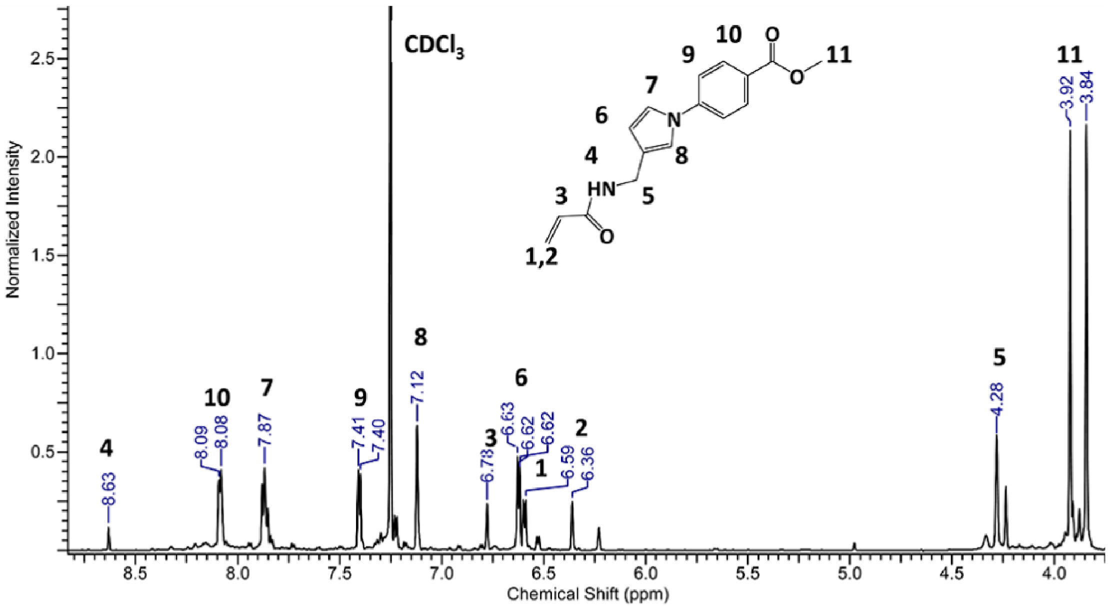


**Figure S12.** $^1$H-NMR spectrum of compound **5.** Some impurities signals from starting material such as (compound **4**–$NH_3$) and solvents can be seen. The estimated relative percentage purity of this compound was found to be about 88% by moles.

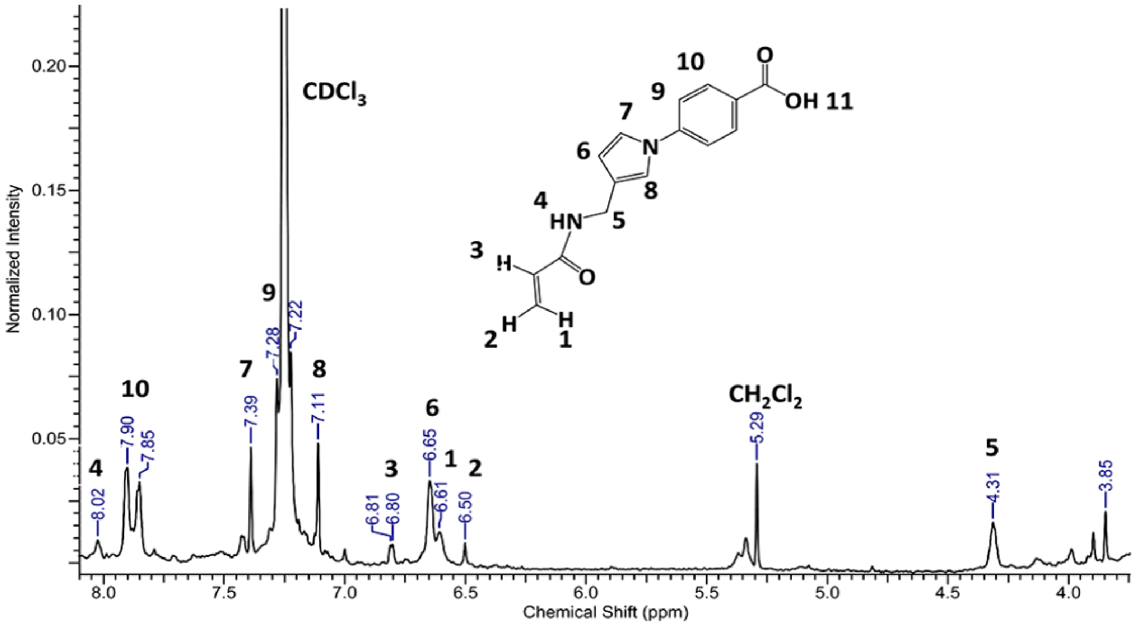


**Figure S13.** $^1$H-NMR spectrum for compound **6**. Some impurities signals from starting material such as such as (compound **4**–$NH_3$) and solvents can be seen. Reduction of peak size at 3.85 ppm demonstrate hydrolysis of compound **5**. The estimated relative percentage purity of this compound was found to be about 90% by moles.

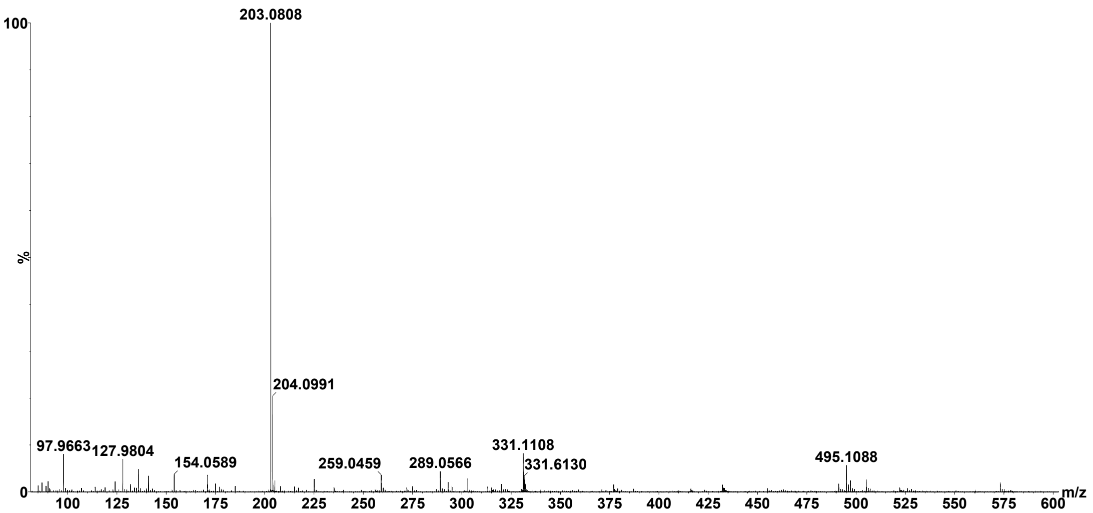


**Figure S14.** HRMS spectrum for compound **7** or RK464 in methanol.

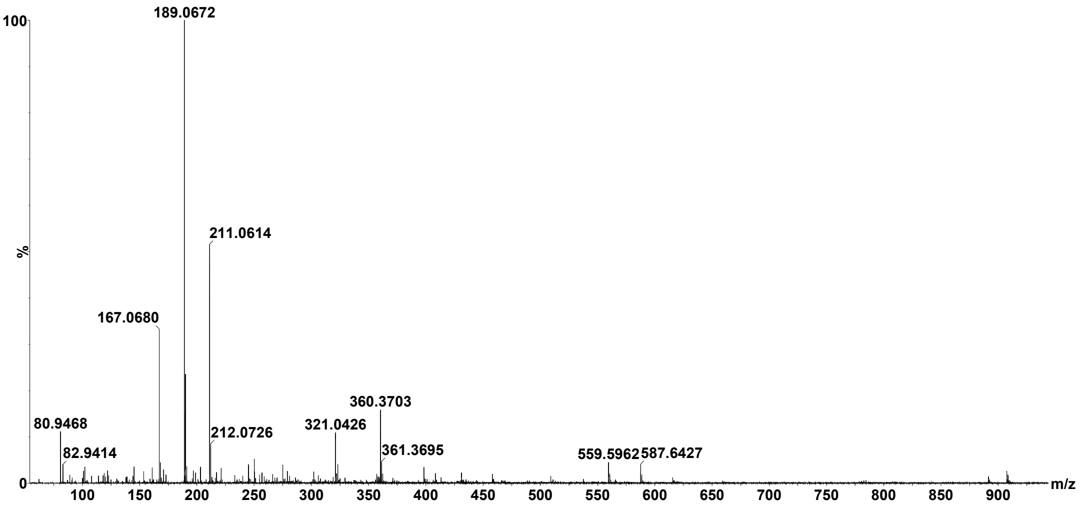


**Figure S15.** HRMS spectrum for compound **8** or RK465 in methanol.

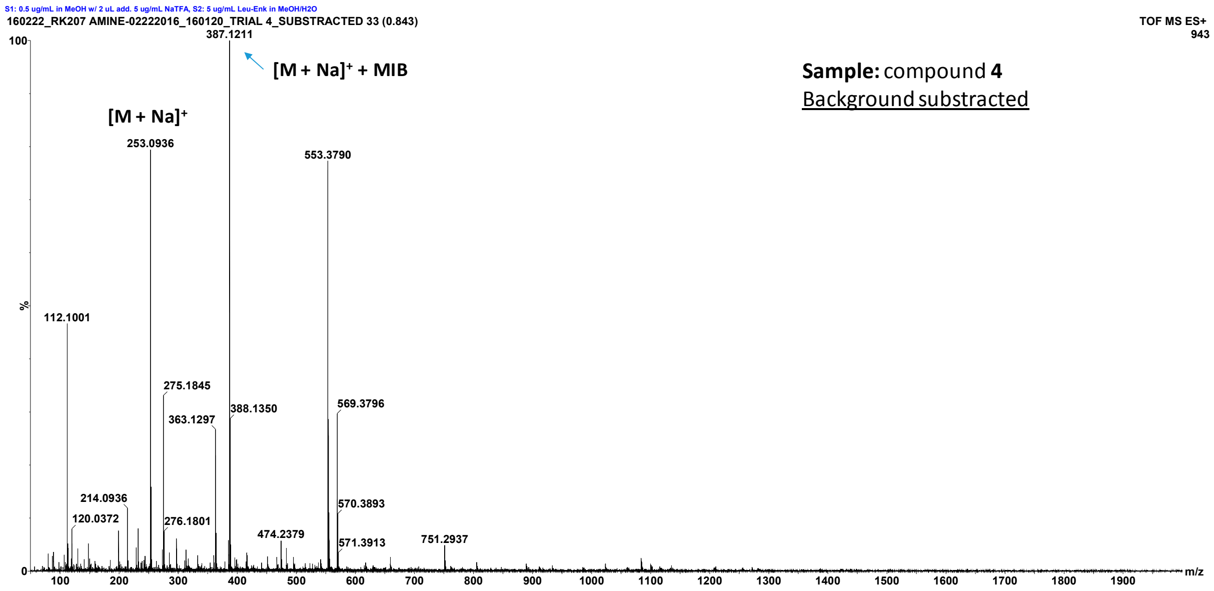


**Figure S16.** HRMS for compound **4**.

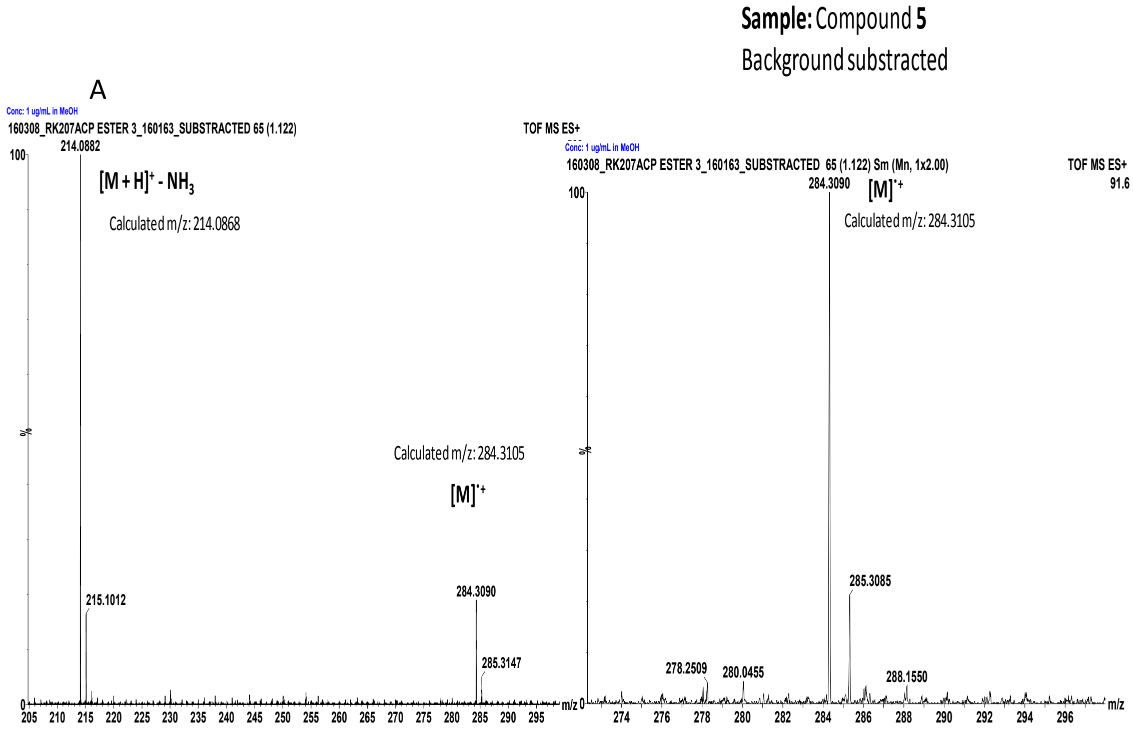

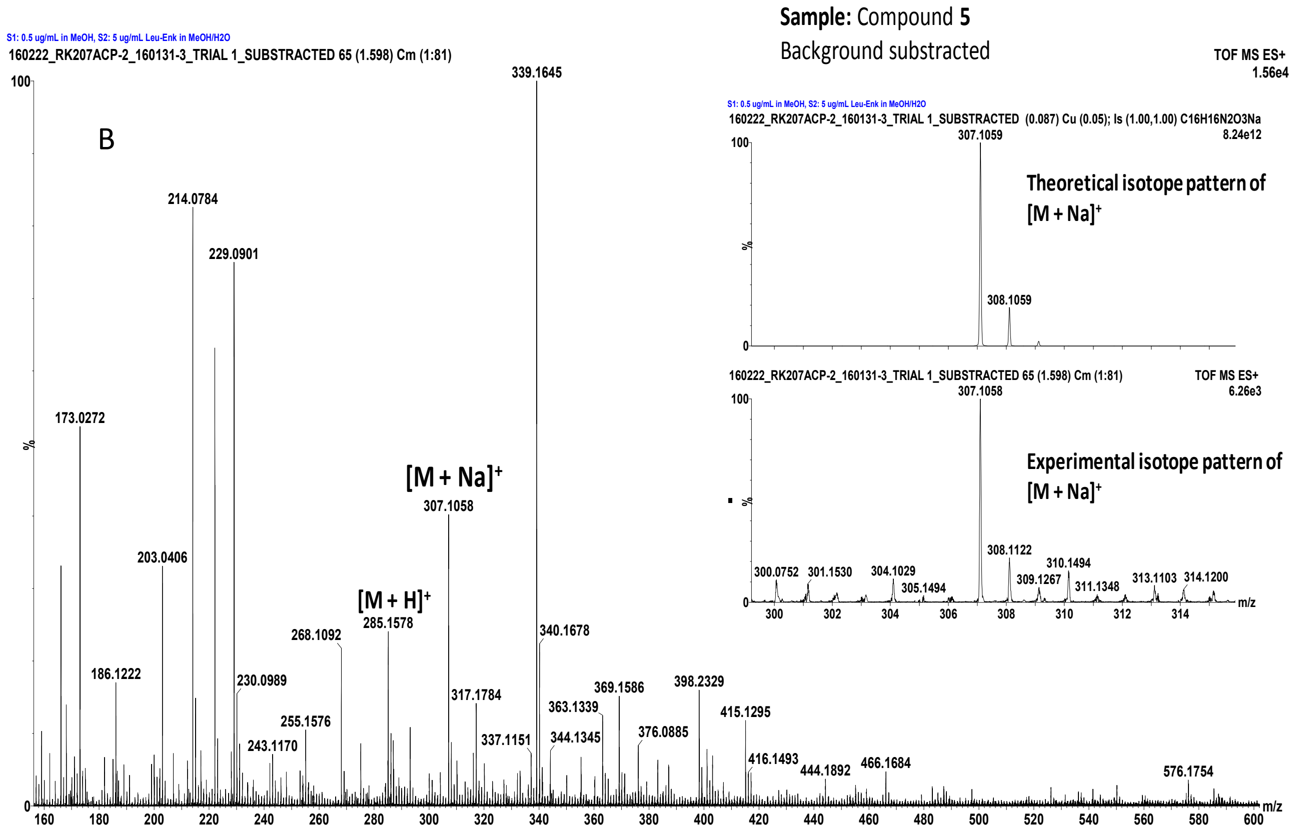


**Figure S17. (A)** HRMS for compound **5**. It contains little impurities, especially (compound **4**–$NH_3$), and **(B)** On further purification of the sample, it get oxidized and forms $[M]^+$, probably because of the presence of a lot of conjugating double bonds in the compound **5.**

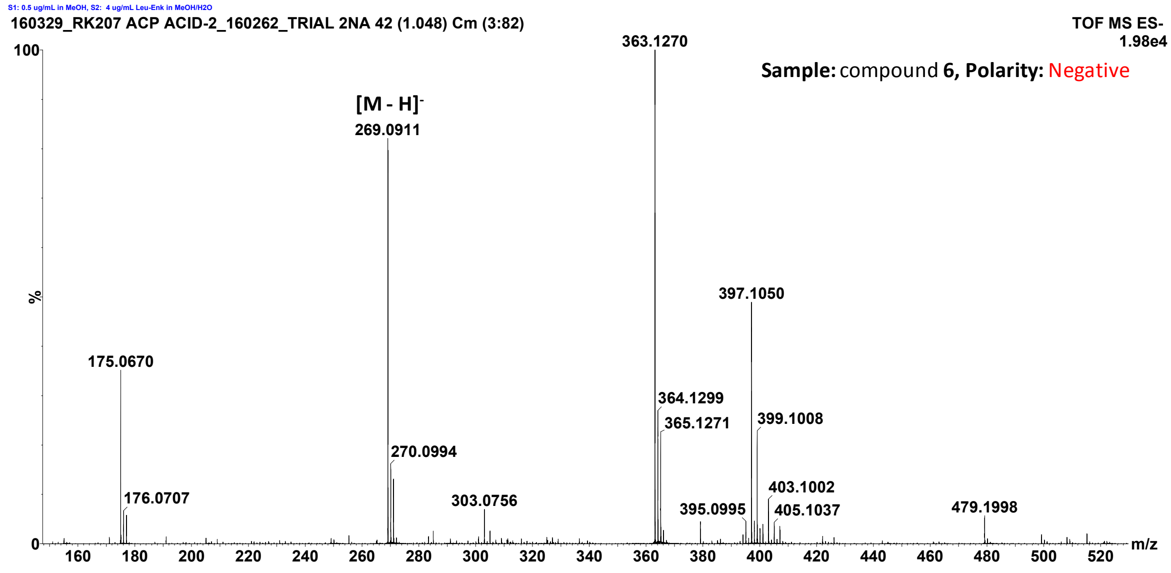


**Figure S18.** HRMS for compound **6** (Negative polarity).

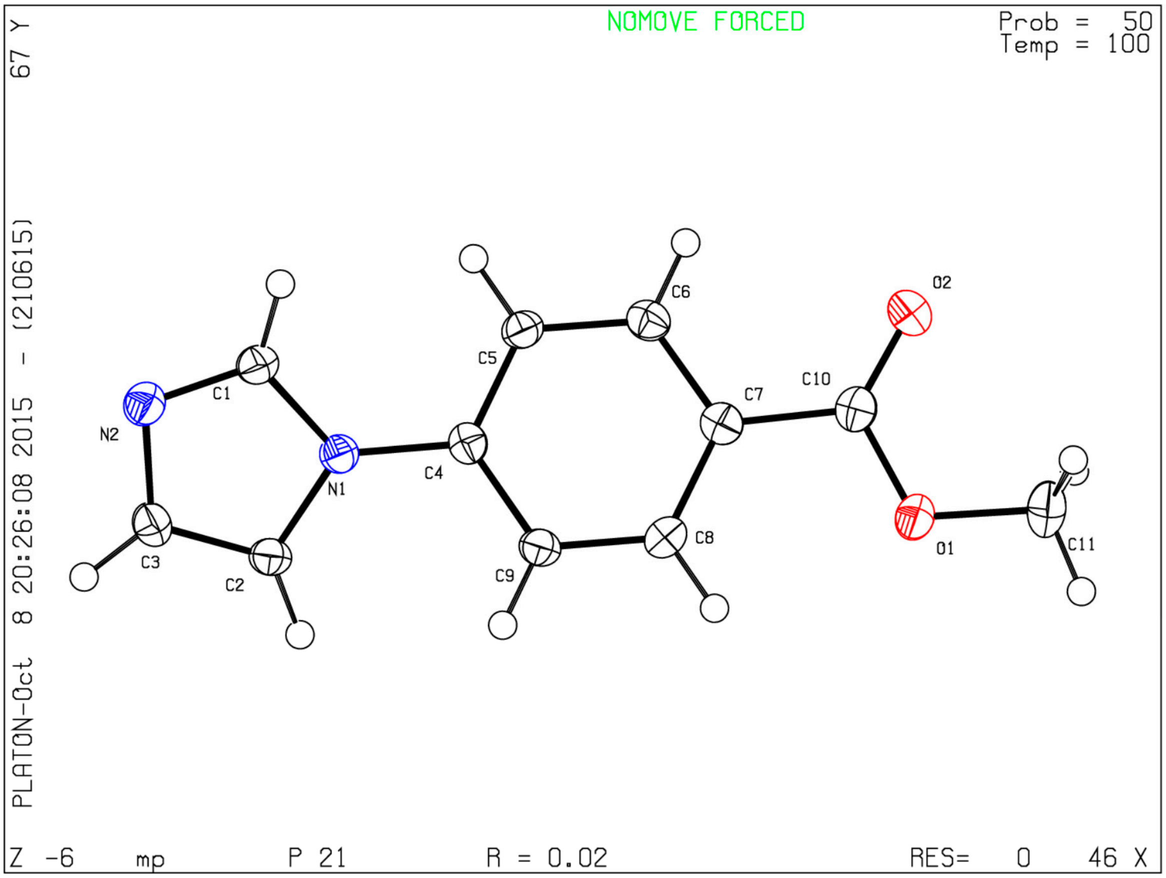


**Figure S19.** X-ray crystallography structure for compound **7** or RK464 **(**CCDC code: CCDC 1471207), with R = 0.2.

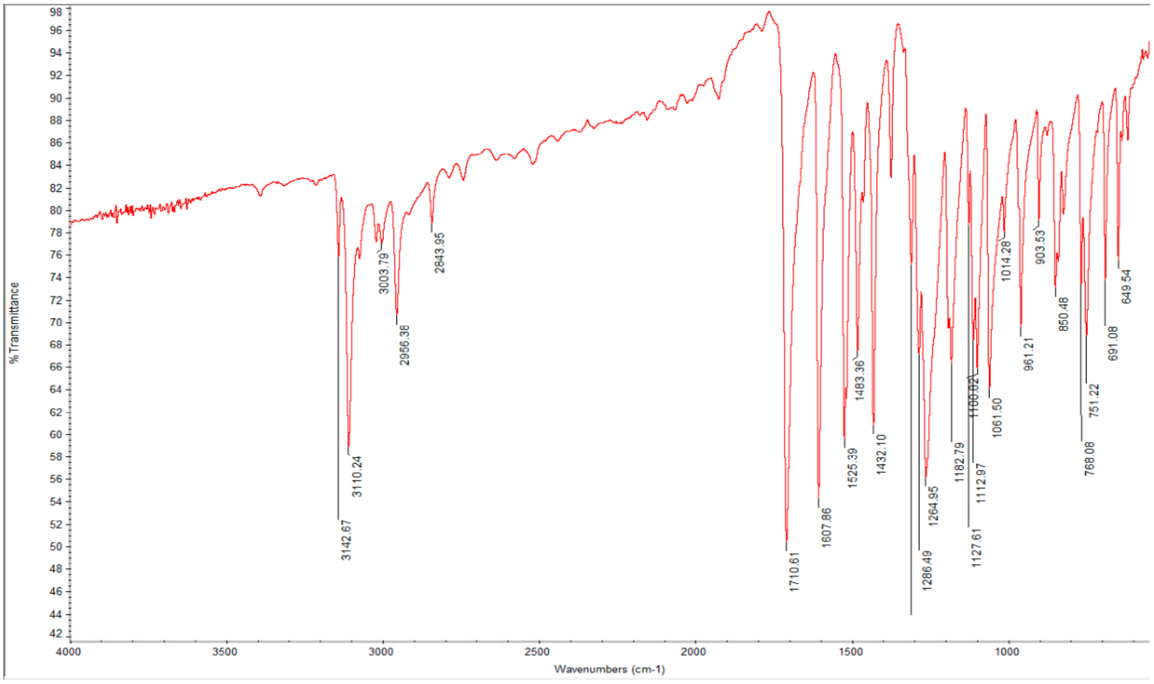


**Figure S20.** IR for methyl 4-(1H-imidazol-1-yl) benzoate or compound **7** or RK464.

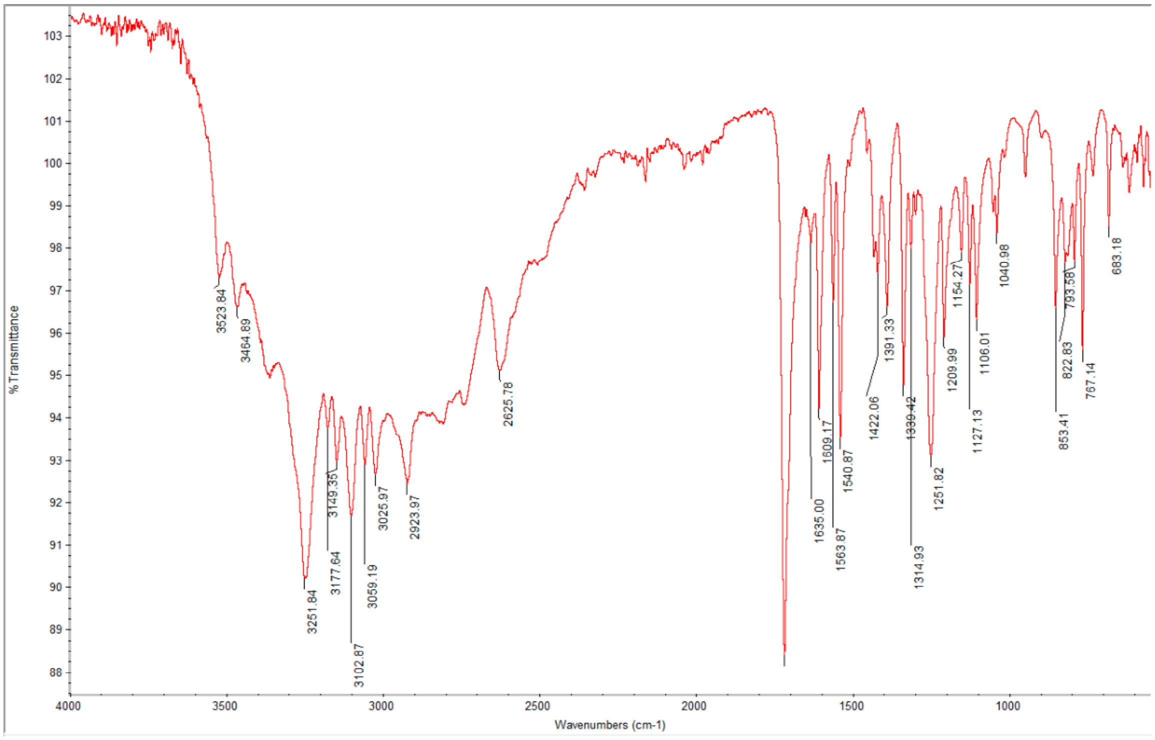


**Figure S21.** IR for 4-(1*H*-imidazol-1-yl)benzoic acid or compound **8** or RK465**.**

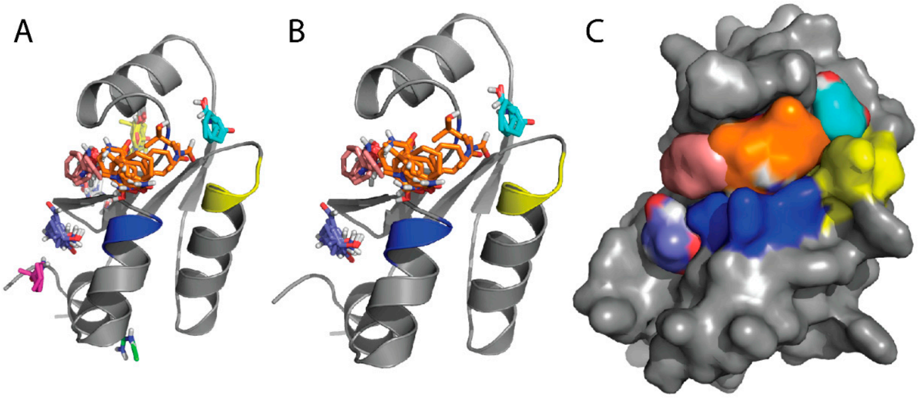


**Figure S22.** Solvent mapping via FTMap. Shifted residues and the active site are colored as in Figure 5 C,D. (**A**) Unfiltered FTMap results. Consensus clusters are rendered as sticks. (**B**) Clusters retained for being in close proximity to shifted residues. Consensus cluster #1 (orange) has the largest number of probe molecules and represents the primary binding site. (**C**) Retained clusters with surfaces rendered. These served to define the binding site for RK207 in molecular dynamics simulations.

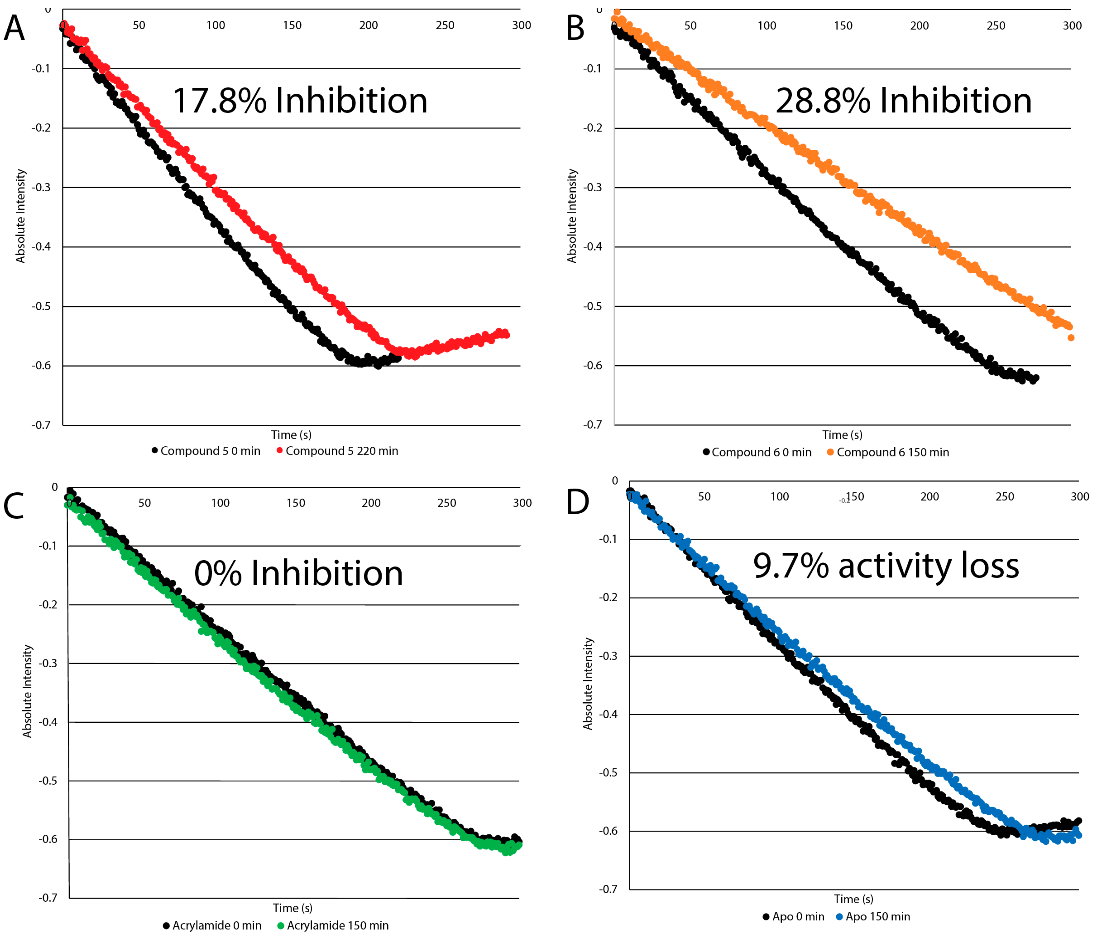


**Figure S23.** BrmGRX activity with lead compounds (**A**) **5** and (**B**) **6**, (**C**) acrylamide, and (**D**) apo with no compound added. Little reduction in activity is seen in the acrylamide and apo samples. In our experience the loss in activity in the apo sample is within normal variation for the HED assay. Loss in activity above this suggests some inhibition due to lead compounds **5** and **6**.

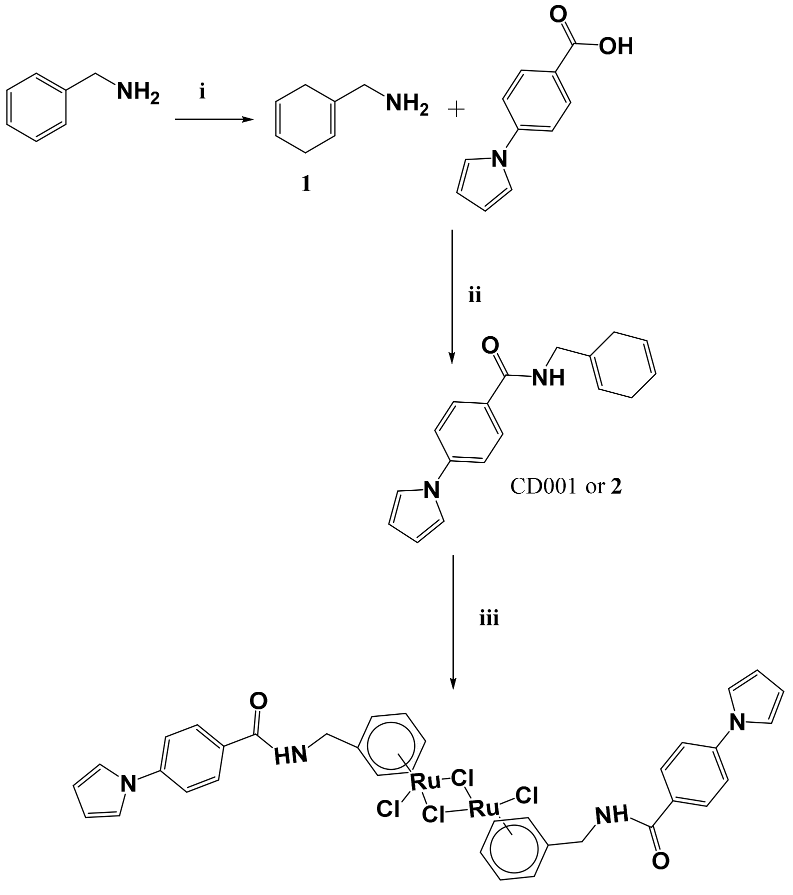


Ru207 dimer or **3**

**(i) Method A: Na(S), $NH_3$(l), EtOH**

**(ii) Method B: EDC, $CH_2Cl_2$, RT**

**(iii) Method C: $RuCl_3$.x$H_2O$, MeOH, 72 hours of reflux**

**Scheme S1.** Synthetic scheme for Ru207 dimer or compound **3** from RK207.Birch reduction was followed to prepare compound **1**, which was later EDC coupled with RK207 to form compound **2**. This amide compound was further metalated with $RuCl_3$.x$H_2O$ to form compound **3**. The change in color of the solution from a dark red to a light yellow-orange confirmed the formation of compound **3**.
